# Season-dependent trait–environment filtering influences interaction networks in mixed-species bird flocks

**DOI:** 10.1098/rspb.2026.1365

**Published:** 2026-10-28

**Authors:** Yong Shen, Zufei Shu, Yingming Zhang, Sergio Estrada-Villegas, Eben B. Goodale, Frederick H. Sheldon, Qing Quan, Fasheng Zou, Fuchen Luan, Hairong Tan, Qiang Zhang

**Affiliations:** ^1^ Guangdong Key Laboratory of Animal Conservation and Resource Utilization, Institute of Zoology, Guangdong Academy of Sciences, Guangzhou 510260, People's Republic of China; ^2^ Guangdong Chebaling National Nature Reserve, Shaoguan 512526, People's Republic of China; ^3^ Department of Biology, Faculty of Natural Sciences, Universidad del Rosario, Bogotá 111711, Colombia; ^4^ Smithsonian Tropical Research Institute, Ancón, 0843-03092, Panama; ^5^ Department of Health and Environmental Science, Xi’an Jiaotong-Liverpool University, Suzhou 215123, People's Republic of China; ^6^ Museum of Natural Science and Department of Biological Sciences, Louisiana State University, Baton Rouge, LA 70803, USA

**Keywords:** ecological networks, environment, functional traits, mixed-species flocks, species interactions

## Abstract

Seasonal variation can strongly influence species interactions, yet its effects on interaction networks remain poorly understood in tropical and subtropical systems. We surveyed mixed-species bird flocks (MSFs) across 20 2 × 2 km grid cells in a subtropical forest in southern China during breeding and non-breeding seasons, quantified seasonal changes in flock composition and network structure, and examined how species traits and environmental factors drive these changes. More species and individuals joined mixed-species bird flocks in the non-breeding season, producing more connected and cohesive networks. During the breeding season, network structure was associated with breeding traits: species with larger clutch sizes showed higher association strength, but overall network connectivity and cohesion declined, suggesting trade-offs imposed by parental care. Network structure in the non-breeding season was related to morphological traits, with smaller species forming more cohesive networks. Environmental effects were also season dependent. Connectivity increased with elevation, forest cover and canopy cover in the breeding season, whereas in the non-breeding season networks were most connected and cohesive at low elevations with high tree species richness and vegetation productivity. Our results demonstrate the importance of seasonal effects on mixed-species bird flocks and highlight the need to maintain mountain connectivity, as communities may rely on different parts of mountains across seasons.

## 1. Introduction

Understanding how biological communities respond to environmental changes is a central theme in ecology [1], especially now that climate change and human disturbances are intensifying globally [2]. Even though the effects of temporal environmental fluctuations on communities are well known [3], the impact of seasonal changes on tropical and subtropical communities is often underestimated because these biomes are climatically more stable relative to other biomes [4]. Yet tropical and subtropical forests can still experience pronounced and ecologically meaningful seasonal variation in climate, especially in temperature and precipitation [5,6]. Therefore, shifts in both temperature and precipitation are expected to have an impact on community structure and composition. For example, seasonality affects the physiological limits of organisms [7] and reproductive phenology [8,9] and influences resource availability in the tropics/subtropics [10–12]. Moreover, communities in tropical and subtropical forests may be more sensitive to future environmental changes because they are adapted to a more stable climate [3]. Investigating how communities respond to seasonal environmental changes can help us predict how they will cope as climate change progresses [13].

Species interactions play a key role in the maintenance of biodiversity and ecosystem function [14], and they help communities respond to environmental changes [15]. A mixed-species bird flock (MSF), defined as ‘a roving group of individuals from at least two species, moving in concert and behaving cohesively while foraging’ [16], is a vital interspecific interaction because it increases foraging efficiency and decreases predation risk [17,18]. MSFs are found worldwide in the non-breeding season in temperate regions and year-round in the tropics [19]. MSFs also respond to environmental change through changes in their density or size or various aspects of their social networks [20–23] (see supplementary material, table S1 for a list of network metrics and their definition and interpretation). For instance, studies looking at how species composition and interspecific associations shift owing to drastic environmental changes have shown that MSFs decrease in size, and the strength of network associations is weakened by increasing human disturbance [24–26]. However, other studies have found that forming MSFs might buffer less intense forms of environmental stress [27,28], by enhancing network connectivity and cohesion with increasing human disturbances [29] and elevational stresses [21]. Overall, MSFs seem to respond in different ways to environmental changes of differing intensities across habitats [23,30,31].

Seasonal changes are not often studied in MSF systems, as MSFs often cease in the temperate breeding season [32]. Nonetheless, studying seasonal changes in tropical and subtropical systems can give insight into how MSFs respond to environmental change [4] and elucidation of the mechanisms that drive MSFs’ formation and membership. For instance, during cold and dry seasons, environmental quality (e.g. vegetative cover and food resources) is relatively poor, and this can trigger an increase in flocking behaviours to improve foraging success and predator defence [33]. This could result in larger MSFs [4,34] and networks with high connectivity and cohesion that buffer environmental stresses [4,21]. In contrast, during the breeding season, relatively abundant resources can lead to diminished benefits of flocking [35]. Further, reproductive demands (nest-building, incubation, feeding nestlings, etc.) could hinder breeding individuals from following MSFs for extended periods of time [36,37]. This should lead to a decrease in MSF size and the connectivity and cohesion of breeding-season foraging networks [4].

Interspecific associations in MSFs are shaped not only by environmental conditions or seasonality but also by the traits of flock members [28,32]. Traits provide mechanistic links between species’ ecological roles and network structure. Morphological traits can influence movement ability, energetic requirements, vulnerability to predators and vertical space use, whereas dietary and foraging traits can affect resource overlap and encounter rates among species [28,38,39]. These processes can determine which species are linked, how strong those links are and whether interactions are concentrated around particular species or distributed more evenly across the network. Consistent with this trait-based view, pairwise association strength in MSFs is related to similarity in body size and foraging behaviours [38], although the effect of body size can depend on flock size [39]. Trait matching also changes across environmental gradients: low-elevation subtropical MSFs tend to include more trait-similar species, whereas high-elevation MSFs show more random composition [21,23]. Similarly, forest succession can modulate trait matching among flocking species, with early- and late-successional forests showing stronger trait similarity than mid-successional forests [40].

Building on this trait-based framework, we still do not know how interspecific associations within tropical and subtropical MSFs shift between breeding and non-breeding seasons. One possibility is that seasonal environmental changes influence the functional trait structure of MSFs, thus affecting interspecific associations [32]. For example, associations during the breeding season might be primarily influenced by breeding traits (e.g. clutch size and nest attendance), because breeding affects species’ resource requirements and behaviours. More specifically, larger clutches require more food [41–43], which in turn, can motivate parents to join MSFs to improve foraging success. Alternatively, other breeding behaviours (e.g. building nests, incubating eggs and feeding nestlings) might lead to networks with less connectivity and cohesion because home ranges of breeders shrink and they spend less time in MSFs [4]. In the non-breeding season, however, MSF participation might be driven more by morphological traits (e.g. body size), since smaller species are more vulnerable to predation and therefore more reliant on flocking [44], leading to more connected and cohesive networks.

In this study, we conducted a systematic survey (across 20 grid cells, each 2 × 2 km) of MSFs in the subtropical Chebaling National Nature Reserve in Guangdong Province, southern China. We surveyed each grid cell twice during the breeding and the non-breeding seasons, measured environmental factors, collected information on species’ traits, and investigated how environmental factors and these traits influenced seasonal differences in MSFs, with a special focus on comparing the seasons in the metrics of their social networks. Specifically, we addressed the following hypotheses: (i) MSFs should be smaller in the breeding season than in the non-breeding season, as manifested by overall lower average species’ association strength (i.e. lower weighted degree (WD); figure 1a), and less connectivity (lower normalized degree (ND) and/or higher skewness of ND) and cohesion (lower clustering coefficients (CCs) and/or higher modularity) of MSF networks (figure 1b). (ii) The network structure of MSFs should depend on the traits of participating species, but these relationships might vary seasonally: network structure should be more closely linked to breeding traits in the breeding season than in the non-breeding season (figure 1c). In the breeding season, species with larger clutch sizes, and thus greater foraging demand, should increase their average association with other species (e.g. have networks with high average WD); yet at the same time, large clutch size could lead to less connected and cohesive networks, as the breeding birds might tend to join MSFs around their nests, without staying long term with the MSFs. In contrast, in the non-breeding season, MSF network structure should be more related to morphological traits such as body size, especially in cold and dry seasons when adult mortality is considerable and anti-predatory behaviour is thus most prominent [38,44]. (iii) We hypothesized that biotic factors (e.g. plant communities) would mediate the impact of abiotic and landscape factors (e.g. elevation) on network-level metrics, and that these environment–metric relationships would exhibit seasonal differences. Specifically, the relationships between environmental factors and networks may be weaker in the breeding season because most MSF members are focused on feeding nestlings when environments are more benign (figure 1d). In contrast, environmental factors could be more important to network structure in the non-breeding season, because harsher environments have a stronger filtering effect on species interactions. Also, to avoid the harsh environment at high altitudes, the most centralized species interactions of MSF networks, which are characterized by well-connected and/or cohesive networks, would be likely to relocate from high elevations in summer to low elevations in winter.

**Figure 1.**
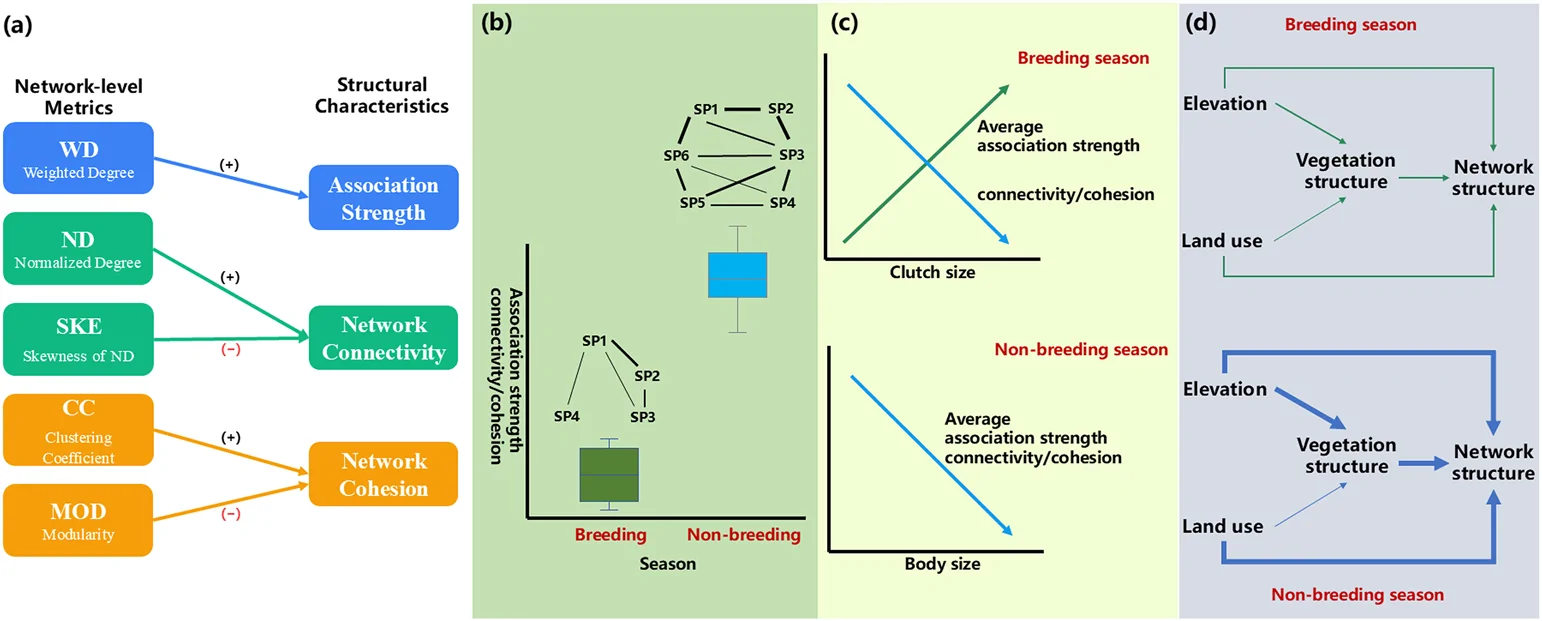
Interpretation of network-level metrics and conceptual illustration of hypothesized changes in mixed-species bird flock (MSF) networks with seasonality. (a) Relationships between the network-level metrics and network structures. Weighted degree (WD): the sum of the simple ratio indexes for each node. Normalized degree (ND): degree is the number of co-occurrences between each species. The degree is normalized for each species by dividing it by the total number of possible connections. Skewness of ND (SKE): skewness of the distribution of normalized degrees. Clustering coefficient (CC): the global weighted clustering coefficient represents the extent to which two species, if both are connected to a third species, are connected to each other. Modularity (MOD): it measures how divided the network is into particular subnetworks. (b) The overall MSF size, network association strength, connectivity and cohesion are expected to be lower in the breeding (wet in our system) season than in the non-breeding (dry) season, because the benefits of flocking decrease when resources are abundant, and participation in MSFs would be reduced by reproductive behaviours. SP1–SP6 indicate different flocking species. (c) In the breeding season, MSF species with large clutch sizes require more food, which might lead to an increase in average association strength, but more feeding behaviours might limit their persistence in MSFs, resulting in less connected and cohesive networks. In the non-breeding season, species with smaller body size require more cooperation to enhance the effectiveness of foraging and defences against predators, especially in the cold and dry winter, leading to stronger association strength and more connected and cohesive networks. (d) The relationships between environmental factors and networks might be weaker in the breeding season because most MSF members are focused on feeding nestlings when environments are more benign. In contrast, environmental factors could be more important to network structure in the non-breeding season because harsher environments have a stronger filtering effect on species interactions.

## 2. Material and methods

### (a) Study site

We conducted this study in the Chebaling National Nature Reserve in Guangdong Province, southern China (24°40′29″–24°46′21″ N, 114°07′39″–114°16′46″ E). The reserve covers approximately 7545 hectares and ranges in elevation from 330 to 1256 m. The climate is subtropical wet and monsoonal. The average temperature is 19.6°C, while the daily temperature ranges seasonally from −5.5°C to 38.4°C. Total precipitation ranges from 1150 to 2126 mm, with a rainy season from March to August and a dry season from September to February. Since the establishment of the reserve in 1981, its vegetation has been well preserved and typical of a subtropical evergreen broad-leaved forest [45]. In 2017, Chebaling established a grid-based wildlife diversity monitoring system that covers the entire reserve [46].

### (b) Bird surveys

We conducted surveys to include the breeding season, which is mainly from March to August, coinciding with the rainy, warm season and the non-breeding season, which includes the other months of the year that are characterized by colder, drier weather. We conducted bird surveys from January to December in 2019. Our study site was divided into 20 grids (each 2 km × 2 km) (supplementary material, figure S1), in each of which we established four survey transects of approximately 1.5 km each. The distance between transects was greater than 600 m, depending on the terrain and accessibility of the grid. Bird surveys were conducted every 2 months by six groups of two to three ornithologists over a period of 3 weeks or less. Surveys were conducted at sunrise and ended before 10.00 in clear weather. During each survey, we recorded bird species and the number of individuals observed within 50 m of the transects on either side, including MSFs, monospecific groups and solitary individuals. All bird surveys were non-invasive observational surveys. No birds were captured, handled or harmed. Fieldwork was conducted with permission from Chebaling National Nature Reserve and complied with local regulations.

### (c) Environmental factors and species traits

We measured six environmental factors in each grid that indirectly reflect bird habitat characteristics and food resources, including both abiotic and landscape and biotic variables [7,11]. Elevation (ELE) and land use, indicated by the proportion of forested land (PFL), were considered as abiotic and landscape variables; canopy cover (CAN_CO), enhanced vegetation index in summer (EVI_S) and winter (EVI_W), tree species richness (SR_P) and the number of individual trees (IND_P) were included as biotic variables. The proportion of forested land was determined using HSPA-1 imagery acquired by the GF-1 (Gaofen-1) satellite and provided by the Aerospace Information Research Institute, Chinese Academy of Sciences. A higher value indicates a greater proportion of forested land in the grid. Canopy cover was collected by the protected area's staff as part of forest inventories at the forest stand level. Using ArcGIS 10.2, we determined the forest canopy cover by measuring all the forest stands that overlapped that grid and then calculating an area-weighted average. The enhanced vegetation index was derived from de-clouded Landsat-8 remote sensing images of Chebaling in 2017 at a 30 m resolution, for breeding and non-breeding seasons, separately. To gather information on plant communities (SR_P and IND_P), we established 12 20 m × 20 m permanent monitoring forest plots in each grid. All woody trees ≥1 cm of diameter at breast height were identified to species and measured. The total number of species and total number of individuals of trees from these 12 plots within each grid represented the overall plant community per grid and served as a proxy for habitat and food resources for birds. Overall, these six environmental factors were not strongly correlated with each other consistently in the two seasons (supplementary material, tables S2 and S3).

We collected eight traits per bird species to represent their body size, diet, foraging stratum and breeding characteristics (supplementary material, table S4). All trait values were obtained from the dataset of Wang *et al.* [47] and Zhang *et al.* [28]. Because body length, body mass and egg volume were highly correlated with each other (supplementary material, tables S5 and S6 for different species in different seasons), we performed a principal component analysis on these three traits. PC1 (the first principal component) reflects body size, as indicated by high positive loadings of body mass, body length and egg volume in both breeding and non-breeding seasons (supplementary material, figure S2 and table S7). In addition, as almost all the flocking species were open-nesting, we removed nest type in further analysis. All traits were standardized by subtracting the mean and dividing by the standard deviation [48].

### (d) Data analysis

#### (i) Data processing

An MSF was defined as a roving group of birds composed of more than one species of foraging individuals staying within 25 m of one other for at least 5 min [49]. Owing to extensive and frequent surveys, we obtained a very large dataset. The records of all birds detected included 157 species and 53 689 individuals. A total of 210 MSFs were observed, including 33 species. To start the analyses, we divided the survey data into two parts: breeding season (March to August, 114 MSFs) and non-breeding season (other months, 96 MSFs) [50]. To ensure appropriate sample sizes (number of MSFs) to construct reliable networks for each grid, only grids that included four or more MSFs within a single season were selected, and an interspecific association network was constructed for each season in each grid. Overall, 9 and 12 networks were constructed for the breeding and non-breeding season, respectively. Subsequent analysis was conducted on these networks.

#### (ii) Flocking frequency and propensity, and MSF sizes

We first calculated flocking frequency and propensity for each species and season (species level, by combining MSF data in all grid cells in each season). Flocking frequency is defined as the number of MSFs containing a given species divided by the total number of MSFs, and flocking propensity was calculated by summing the total number of individuals of a given species during surveys and calculating what percentage of those individuals occurred in MSFs [34,51]. To determine whether flocking frequency and propensity differed between seasons, we conducted linear mixed-effects models, using the ‘lme4’ package, with season as the independent variable, and species as a random effect. Pairwise multiple comparisons were conducted using the R package ‘emmeans’.

MSF size was measured by the number of species and individuals per MSF. We determined differences in the number of species and individuals in MSFs between breeding and non-breeding seasons (*testing hypothesis #1*), also using linear mixed-effects models, with season as the independent variable, and grid cell as a random effect. Pairwise multiple comparisons were also conducted in the same manner as above.

#### (iii) MSF association networks and their metrics

We compared MSF structure between the breeding and the non-breeding seasons using network analysis. These analyses assumed that any species occurring in an MSF was connected with the rest of the species in that MSF [24,29]. First, we calculated a simple ratio index (SRI) for species pairs using species co-occurrence data. Then, we constructed weighted networks using SRIs for each grid in each season to determine interspecific association strength [20]. The weighted networks also incorporate a metric for the strength of the associations (i.e. edge weights) between connected species. Networks were constructed with the R package ‘igraph’ [52].

We calculated five network-level metrics to represent network structure (supplementary material, table S1). WD and ND are originally node-level parameters: WD represents the summed SRI-weighted associations of each species. ND represents the proportion of realized associations relative to the maximum possible number of associations. Thus, WD reflects association strength, whereas ND reflects network connectivity. At the network level, we summarized these metrics by taking the mean across all species in a network. Network density also measures connectivity, but when using SRI to construct a weighted network, density is numerically equal to ND, so we only report ND. Higher WD corresponds to stronger association strength among species, whereas higher ND and lower skewness of ND (SKE) indicate higher network connectivity. Higher CC and lower modularity (MOD) indicate greater network cohesion, reflecting the extent to which MSFs are partitioned into modules (as defined via random walks in the package ‘igraph’). To determine whether these metrics differed between seasons (*testing hypothesis #1*), we used linear mixed-effects models with season as the independent variable and grid cell as a random effect, and pairwise multiple comparisons were conducted as above. For species-level network roles, we calculated closeness centrality, z-score and participation coefficient [30,53].

In the breeding-season analysis, there were three species that were migrants and were found in MSFs in early spring, before they left the region. We kept these species in the network analyses for the breeding season, as their presence affected the other species’ network metrics.

#### (iv) Null models for MSF networks

We generated 9999 null MSF datasets by permuting the observational data while preserving species richness and association incidence per MSF [54]. Weighted networks and the five network-level metrics were recalculated for each null dataset, and observed values were considered significantly different from null expectations when they fell outside the central 95% of the null distribution [30]. Permutations were performed using the ‘asnipe’ package [55].

#### (v) Relationships between network-level metrics and species’ traits in each season

To identify traits of bird species in different grid cells and seasons (*testing hypothesis #2*), we calculated community-weighted mean (CWM) trait values per grid cell and per trait for each season. We used bird species abundance (MSF participants) in each network to calculate CWM values as follows: CWM = $\sum_{i\;=\;1}^{n}P_{i}\times trait_{i}$, where *n* is the species number found in a given dataset, *P_i_* is the relative abundance of species *i* in that grid cell and trait*_i_* is the trait value of species *i* [56]. CWM was calculated with the package ‘FD’ [57]. We examined relationships between CWM traits and network-level metrics using separate regression models for each trait–metric combination. WD was analysed using Gamma GLMs (generalized linear models) [23], ND, CC and MOD using beta regression [58], and SKE using Gaussian GLMs. To test seasonal differences, we included trait × season interactions in partial regression models.

In the CWM analysis, we did not include the three migratory species, as we did not want them to influence the breeding analysis (i.e. the characteristics of their breeding did not influence this community).

#### (vi) Impacts of environmental factors on network-level metrics

We used structural equation modelling (SEM) to examine how abiotic and landscape environmental factors (elevation and proportion of forested land) influence biotic environmental factors (canopy cover, enhanced vegetation index, tree species richness and number of individual trees) and how both abiotic and landscape and biotic factors, in turn, affect network-level metrics (*testing hypothesis #3*). In each SEM, abiotic and landscape variables were specified as exogenous predictors, biotic variables as intermediate mediators and network-level metrics as endogenous response variables. We initially included all plausible direct paths from abiotic/landscape to biotic variables and from abiotic and landscape and biotic variables to network-level metrics. Paths were then removed sequentially if their exclusion did not decrease model fit (AIC (Akaike information criterion) no longer decreased) [59]. Separate SEMs were constructed for the breeding and non-breeding seasons using the ‘piecewiseSEM’ R package [60]. To quantify the combined influence of each predictor, we calculated standardized total effects by summing the direct and indirect path coefficients from each environmental variable to each network-level metric, capturing both direct effects and effects mediated through biotic variables.

## 3. Results

### (a) Flocking frequency and propensity in breeding and non-breeding seasons

We observed 29 and 31 flocking species and 3121 and 4286 flocking individuals in 114 breeding and 96 non-breeding-season MSFs. The mean number of MSFs per grid cell (MSF density) was higher in the breeding season (supplementary material, table S8).

Flocking frequency was quite stable across seasons. Huet's fulvetta (*Alcippe hueti*), chestnut bulbul (*Hemixos castanonotus*) and grey treepie (*Dendrocitta formosae*) flocked with high frequency in both seasons (supplementary material, table S9). However, the flocking propensity of other species differed between seasons. In the breeding season, migratory warblers (Phylloscopidae) flocked with high propensity, including dusky warbler (*Phylloscopus fuscatus*), Pallas's leaf warbler (*Phylloscopus proregulus*) and yellow-browed warbler (*Phylloscopus inornatus*). In addition, Japanese white-eye (*Zosterops japonicus*) and mountain tailorbird (*Phyllergates cucullatus*) also flocked commonly in the breeding season (propensity ≥0.50). In comparison, in the non-breeding season, warblers reduced flocking propensity. Further, in the non-breeding season, species from a wide variety of different taxa showed higher flocking propensity (≥0.50), including large woodshrike (Prionopidae, *Tephrodornis gularis*), chestnut-crowned warbler (Phylloscopidae, *Seicercus castaniceps*), red-billed leiothrix (Timaliidae, *Leiothrix lutea*), light-vented bulbul (Pycnonotidae, *Pycnonotus sinensis*) and white-bellied yuhina (Timaliidae, *Erpornis zantholeuca*). However, overall flocking frequency and propensity did not differ significantly between breeding and non-breeding seasons (supplementary material, figure S3 and table S10).

### (b) Comparison of MSF sizes and network-/species-level metrics between breeding and non-breeding seasons

The average number of species and average number of individuals in MSFs were significantly lower in the breeding season than in the non-breeding season (*n* = 210; *β* = −0.481, *p* = 0.044 and *β* = −23.200, *p* < 0.001, respectively; figure 2 and supplementary material, table S10). MSF networks in the non-breeding season were more connected and cohesive than in the breeding season. Specifically, the average weight that is related to association strength was significantly higher in the non-breeding season than in the breeding season (*n* = 21; *β* = −1.250 and *p* = 0.024; figure 3a and supplementary material, table S10). Network connectivity was higher in the non-breeding season than in the breeding season: ND, which is positively related to connectivity, was significantly higher (*β* = −0.078 and *p* = 0.048; figure 3b and supplementary material, table S10), whereas skewness of ND, which is negatively related to connectivity, was significantly lower (*β* = 0.902 and *p* = 0.007; figure 3d and supplementary material, table S10). Finally, the CC that is positively related to network cohesion was significantly higher in the non-breeding season than in the breeding season (*β* = −0.247 and *p* = 0.001; figure 3c and supplementary material, table S10). The observational network-level metrics deviated from expected null models, with the observed values showing a significant difference (*p* < 0.05) for the majority of networks, especially for the WD, ND and CC (red circles in figure 3). These findings suggest that social interactions among flocking species varied across grid cells and that the observed patterns were not merely the result of species associations owing to shared habitat preferences.

**Figure 2.**
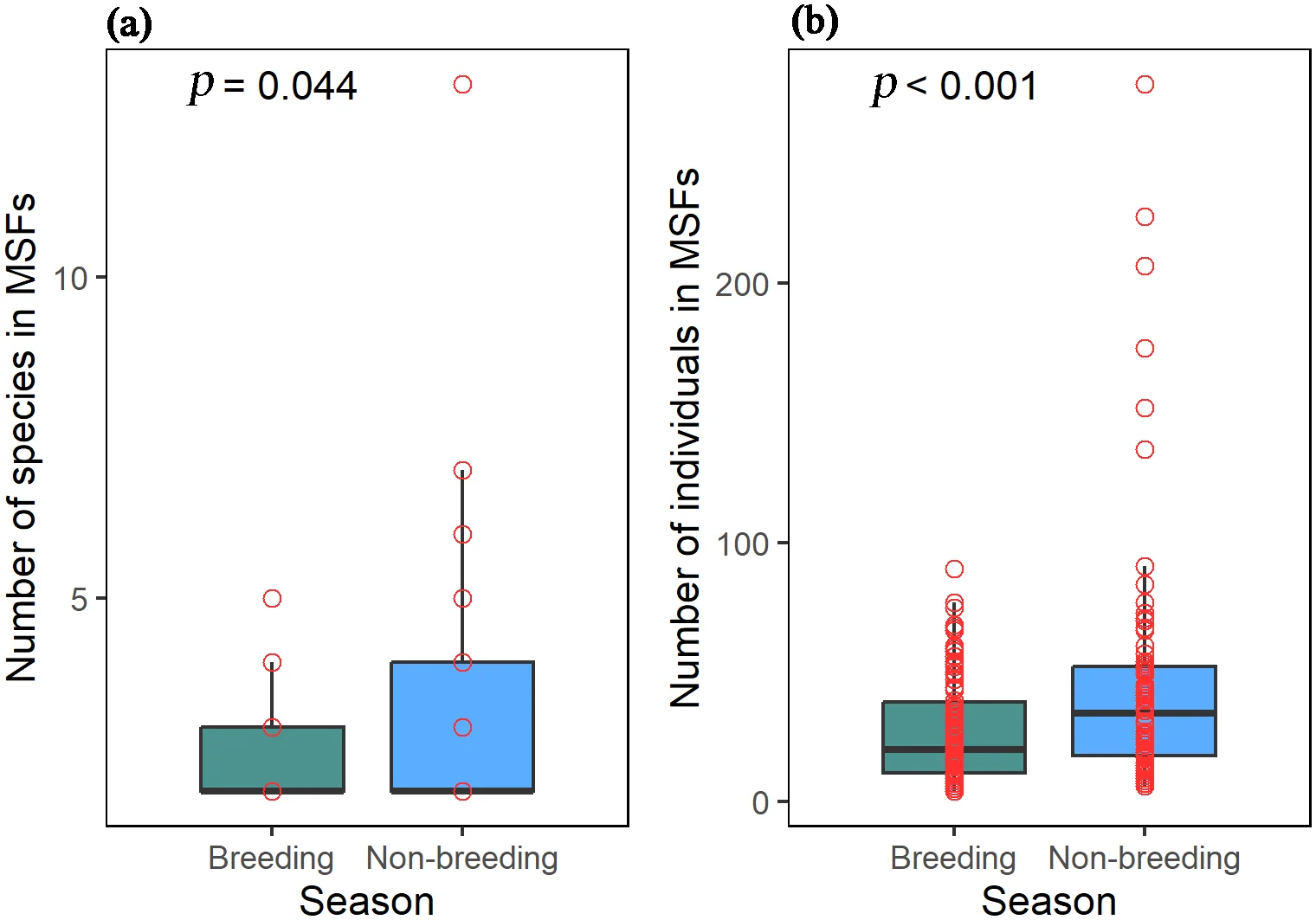
The number of species (a) and number of individuals (b) in mixed-species bird flocks (MSFs) were significantly lower in the breeding season than in the non-breeding season. *p*-Values were obtained from linear mixed-effects models, with season as the independent variable and grid cell as a random effect.

**Figure 3.**
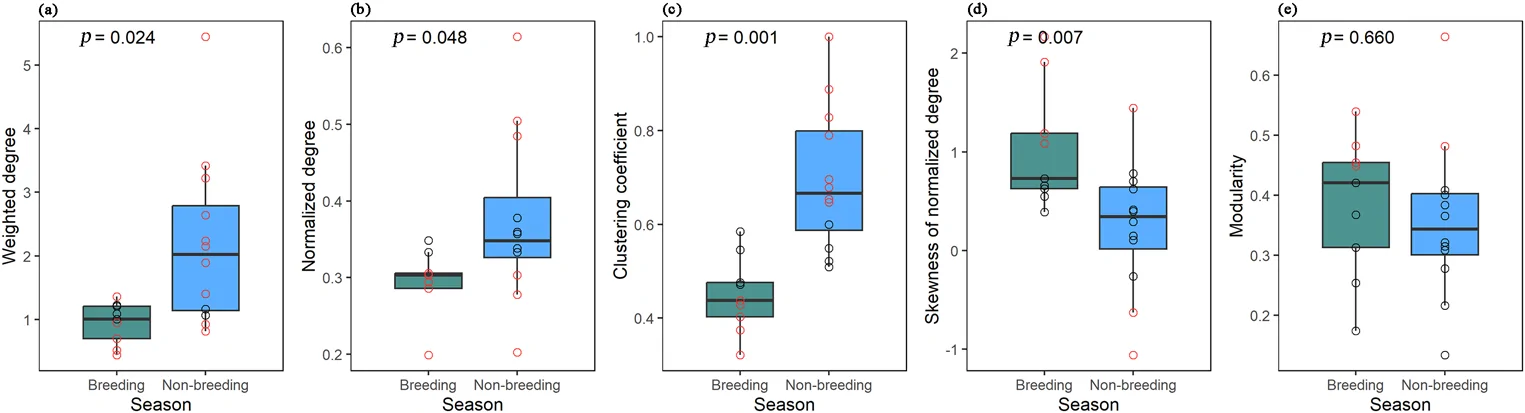
Differences in network-level metrics of mixed-species bird flocks (MSFs) between the breeding and the non-breeding seasons. (a) Weighted degree, (b) normalized degree (ND), (c) clustering coefficient, (d) skewness of ND and (e) modularity. *p*-Values were obtained from linear mixed-effects models, with season as the independent variable and grid cell as random effect. The red circles indicate that observed values significantly depart from 9999 values generated randomly (*p* < 0.05), while black circles indicate that the observed values are not significant.

The roles of species in MSFs also changed seasonally. In the breeding season, Huet's fulvetta and chestnut bulbul had the highest centrality values in five and four networks, respectively (supplementary material, table S11). These two species were also well connected with other species within and outside their modules (high participant coefficients and *z*-scores). In comparison, in the non-breeding season, a greater variety of species had high closeness centrality in the MSF networks, including grey treepie and rufous-capped babbler (*Stachyris ruficeps*) (supplementary material, table S12). Grey treepie, chestnut bulbul and black bulbul (*Hypsipetes leucocephalus*) were important in connecting other species within their modules in the non-breeding season.

### (c) Relationships between CWM traits and network-level metrics

In the breeding season, clutch size was the most important trait influencing network structure (figure 4a–c and supplementary material, table S13), as few other traits showed significant relationships with network-level metrics. Networks with higher CWM clutch sizes had higher WD and skewness of ND (*n* = 9; *β* = 2.649, *p* = 0.021 and *β* = 3.442, *p* = 0.045, respectively; figure 4a,b), and larger clutch sizes were associated with higher modularity (*β* = 3.496, *p* = 0.001; figure 4c), indicating stronger average associations but increased heterogeneity and reduced connectivity and cohesion in networks dominated by species with larger clutch sizes. In addition, ND declined with increasing CWM stratum, suggesting that species occupying higher vertical strata had lower average network connectivity (supplementary material, figure S4).

**Figure 4.**
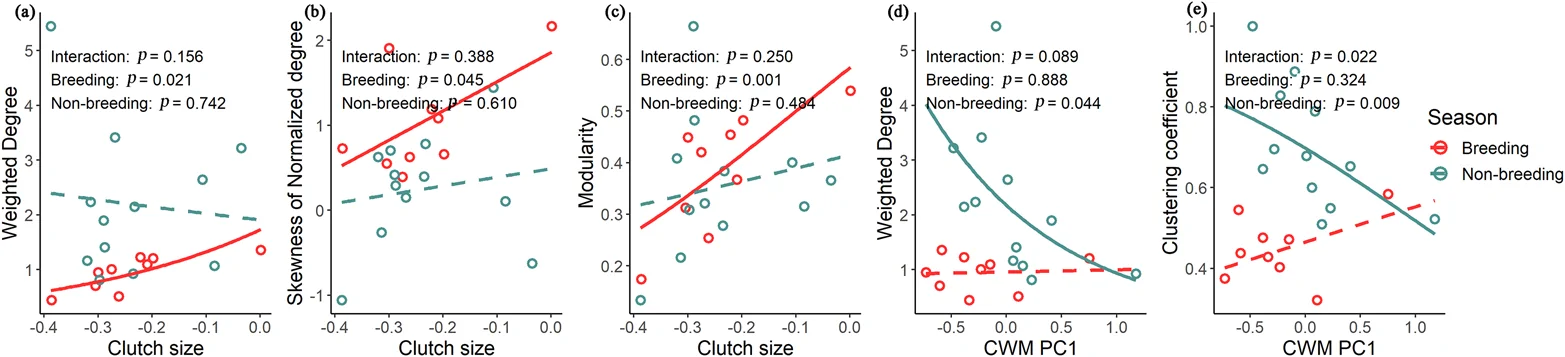
Relationships between community-weighted mean (CWM) trait values and network-level metrics of mixed-species bird flocks (MSFs) across breeding and non-breeding seasons. (a–c) Relationships between clutch size and WD, SKE and MOD, respectively; (d,e) relationships between PC1 (for body length, body mass and egg volume) and WD and CC, respectively. Lines and *p*-values are derived from partial regression models. Solid lines indicate significant relationships (*p* < 0.05), and dashed lines indicate non-significant relationships. Red lines and points represent the breeding season, while green lines and points represent the non-breeding season. Trait values were standardized.

In the non-breeding season, CWM PC1, representing larger body size, was negatively associated with WD and CC (*n* = 12; *β* = −0.842, *p* = 0.044 and *β* = −1.024, *p* = 0.009, respectively; figure 4d,e and supplementary material, table S13). Only the interaction between CWM PC1 and season for CC was significant (*n* = 21; *β* = −0.543 and *p* = 0.022; figure 4e and supplementary material, table S14), indicating that the relationship between body size and network cohesion differed between seasons. Specifically, the slope was negative in the non-breeding season, whereas it was weak or absent in the breeding season. Thus, smaller-bodied species contributed more strongly to cohesive networks mainly during the non-breeding season. Overall, these results highlight season-specific trait effects on network structure, with clutch size dominating during breeding and body size composition influencing certain network properties during the non-breeding season.

### (d) Impacts of environments on network structure

The SEMs showed that environmental and biotic variables affected MSF network structure through both direct and indirect pathways, and these pathways differed between seasons (figure 5; supplementary material, tables S15 and S16). In the breeding season, only four environmental variable-network metric relationships showed significant effects. Elevation and proportion of forested land had direct positive effects on ND, while canopy cover acted as an important mediator linking habitat structure to network metrics, particularly WD and skewness (figure 5a). In the non-breeding season, more environmental variable-network metric relationships had significant effects, indicating a stronger environmental influence outside the breeding period. In particular, elevation, proportion of forested land, tree species richness, number of individuals and winter-enhanced vegetation index were associated with multiple network properties, including WD, CC, skewness and modularity (figure 5b).

**Figure 5.**
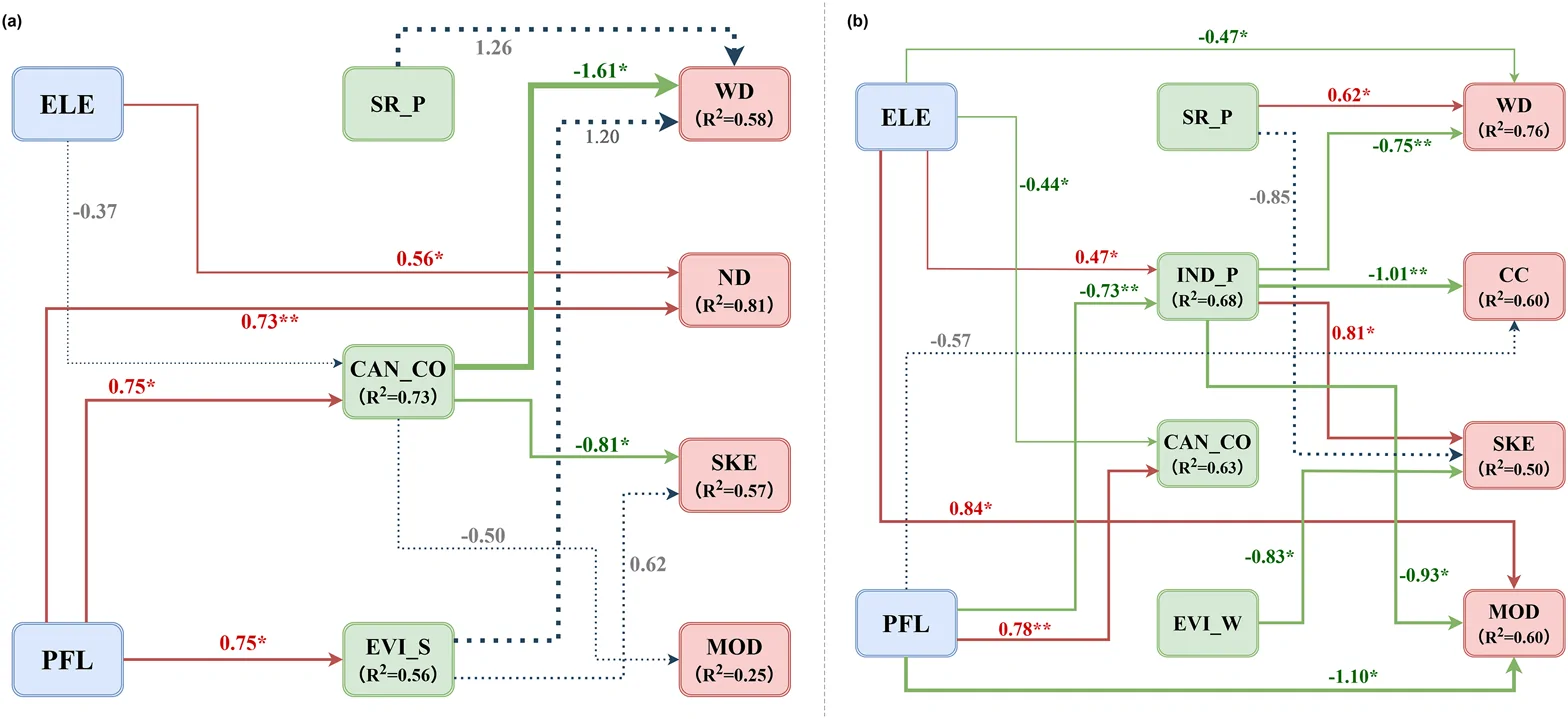
Optimal structural equation model (with the lowest AIC) relating network-level metrics (red boxes), biotic (green boxes) and abiotic/landscape (blue boxes) environmental factors in the breeding (a) and non-breeding seasons (b). Red and green lines indicate significant positive and negative relationships, respectively. Grey dotted lines indicate non-significant relationships. Thicker lines correspond to stronger relationships, numbers along the arrows are path coefficients, and numbers in brackets are *R*^2^ values. ELE, elevation; PFL, proportion of forested land; SR_P, species richness of trees; IND_P, number of individual trees; CAN_CO, canopy cover; EVI, enhanced vegetation index in summer (EVI_S) or in winter (EVI_W); WD, weighted degree; ND, normalized degree; CC, clustering coefficient; SKE, skewness of normalized degree; MOD, modularity. * *p* < 0.05; ** *p* < 0.01. In the final optimized SEMs, no retained paths were associated with CC in the breeding-season model or ND in the non-breeding-season model; these metrics are therefore not shown.

The standardized total effects further clarified the relative importance of predictors by combining their direct and indirect effects (supplementary material, figure S5). In the breeding season, total effects were mainly concentrated on WD and ND (supplementary material, figure S5a). Elevation had positive total effects on both WD and ND, indicating stronger associations and greater connectivity at higher elevations. The proportion of forested land positively affected ND but had negative total effects on WD, skewness and modularity, partly through its influence on canopy cover. Canopy cover showed the strongest negative total effect on WD and also reduced skewness and modularity. In contrast, plant species richness and the summer-enhanced vegetation index had strong positive total effects on WD.

In the non-breeding season, total effects were distributed across more network metrics (supplementary material, figure S5b). Elevation negatively affected WD and CC but positively affected skewness and modularity. The proportion of forested land and tree species richness increased WD and reduced skewness or modularity, suggesting stronger and more cohesive networks in forested and tree-rich habitats. The number of individuals had negative total effects on WD, CC and modularity, but a positive effect on skewness. The winter-enhanced vegetation index also reduced skewness. Overall, these results indicate that environments influenced flock network structure through season-specific pathways, with breeding-season effects being more concentrated on association strength and connectivity and non-breeding-season effects being more broadly distributed across multiple aspects of network structure.

## 4. Discussion

We investigated how environmental factors and species traits regulated seasonal differences in the network structure of MSFs. Overall, we found that MSFs had larger average sizes and formed more connected and cohesive networks in the non-breeding season than in the breeding season. As predicted, clutch size explained network structure in the breeding season, whereas morphological traits explained network structure in the non-breeding season. Network structure of MSFs was more dependent on environmental factors in the non-breeding season than in the breeding season. Our results provide important context for understanding the conditions under which MSFs form across space and time. For instance, 62% of studies assessing the structure of MSFs have only been conducted in one season [32], and habitat effects could be misleading if seasonality is not integrated [4]. We concur with Goodale *et al.* [61] that seasonal differences in MSFs require greater study, and we believe that our results provide new evidence on how MSFs reassemble to cope with seasonal differences in local environments.

### (a) MSF sizes were larger in the non-breeding season

Few studies have shown seasonal differences in MSFs and those that have mainly focused on describing MSF sizes. For example, MSFs in Borneo had the largest sizes in the non-breeding (dry) season [62,63], and those in southern Brazil decreased in size during the breeding season [35]. In the Atlantic Forest, also in Brazil, seasonality influenced the numbers of individuals, but not species richness [64]. Flocking frequency also seems to change between seasons according to forest strata. Zou *et al.* (2011) found that canopy birds had higher flocking frequency in the breeding (wet) season, whereas understorey birds had higher flocking frequency in the non-breeding (dry) season [65].

Consistently, Zhang *et al.* found that the average number of species and individuals was higher in the non-breeding (dry) season than in the breeding (wet) season in a subtropical forest located near our study site in southern China [34]. An interesting finding of this study is that MSF density (number of MSFs per unit area) was higher during the breeding season, 12.67 MSFs/grid, compared with 8.00 MSFs/grid during the non-breeding season. This suggests that the breeding season has smaller but more numerous MSFs, which conceivably have smaller home ranges. Seasonality, thus, can have a profound effect on the size and spatial requirements of MSFs [64], probably reflecting both the abundances of resources [35], and the needs and requirements of reproduction.

### (b) MSFs built more connected and cohesive networks in the non-breeding season

Consistently, in the breeding (wet) season, species built less connected and cohesive networks compared with the non-breeding (dry) season (*hypothesis #1*; figure 3). These results illustrate that breeding behaviours of flocking species such as building nests, incubating eggs and feeding nestlings [16,36,37,66], may physically and temporally constrain species from participating in MSFs during the breeding season [4]. Another reason for such differences in structure might be related to the costs and benefits of joining an MSF. When resources are abundant (e.g. high arthropod biomass and abundance) and/or predation risk is low, species are likely to benefit more from foraging alone or in a conspecific group than in an MSF [35,67], especially when they have a nest or offspring to visit. In contrast, MSF participation should become increasingly important in providing foraging and anti-predation benefits when resource availability is low and/or predation risk is high (e.g. in the dry season) [35,38,68].

Unlike our study site, many Amazonian MSF members breed in the dry season. They found that Amazonian MSFs included more species but formed less complex and cohesive networks in the breeding (dry) season than in the non-breeding (wet) season [4]. They inferred that MSFs that range over a wider area would maximize opportunities for more species to temporarily join MSFs in the dry (breeding) season, increasing species richness in MSFs. However, constraints inherent to the breeding season and changing food availability would also decrease the ability of those species and individuals to persist over time in MSFs, thereby weakening the MSF networks. Hence, it is likely that the network structure of the breeding season was primarily affected by reproductive behaviours, but not by seasonal environmental changes. Network structure has been reported as being weak during the breeding season, whether this occurred in the resource-rich wet season (figure 3) or the resource-poor dry season [4].

### (c) Clutch size and body size seasonally determine MSF network structure

Previous studies have shown that integrating network theory and trait-based ecology can uncover interspecific associations within MSFs [21,40]. Interestingly, here we found that different species traits shaped the network structure in different seasons (figure 4). These trait-based associations may reflect shifts in resource requirements throughout the year. During the breeding season, species with larger clutch sizes are reported to have higher food requirements [41–43], which may drive them to join MSFs to forage more efficiently. At the same time, these species might not be able to move far from their nests, which may be related to denser, but less far-ranging MSFs as discussed above. The small sizes of these MSFs, and the shorter times in which birds stay with them, could lead to less connected and cohesive networks (figure 4 and Rutt & Stouffer [4]). It is also noteworthy that we earlier discussed differences in the flocking frequency between canopy and understorey bird species: higher flocking frequency occurred in the wet season for canopy birds, but in the dry season for understorey birds in a tropical forest in Hainan, southern China [65]. However, this previous result slightly differs from our findings in this subtropical forest, where understorey MSFs had relatively higher association strength and connectivity, compared with birds in higher strata, during the breeding season (wet season).

In contrast, in the non-breeding season, morphological traits (small body size) appeared to play a more important role in influencing MSF structure, consistent with previous findings that smaller species are more vulnerable to predators and thus more likely to join MSFs [38,44]. The relationship between body size and network cohesion differed between seasons, with smaller-bodied species contributing more strongly to cohesive networks during the non-breeding season. Previous studies have reported that smaller species may experience higher predation risk [38,44], which may promote tighter associations in MSFs. These findings further illustrate that seasonality should not be ignored and needs to be explicitly integrated when studying MSF networks [4].

### (d) Habitat effects on network structure were more significant in the non-breeding season

The structure of MSFs is expected to change across environmental gradients, because the benefits of flocking are context specific [23]. However, most studies looking at the effects of environmental changes across space on MSF structure have omitted potential seasonal effects [24,26,30,31]. Our results show that MSF network–environment relationships can be quite different between the breeding and the non-breeding seasons. Overall, relationships between network structure and environmental factors were weak in the breeding season, but stronger in the non-breeding season. Network connectivity increased (indicated by higher ND and lower skewness) in higher elevation sites and with a high proportion of forested land and canopy cover during the breeding season (figure 5), consistent with Shen *et al.* [21]. In the non-breeding season, however, there were more influential factors: MSFs formed relatively strong networks (indicated by higher CC and lower skewness and modularity) at lower elevation sites and with a high proportion of forested land, tree species richness, EVI and a lower number of trees (figure 5).

Other studies have also evaluated how MSF structure changes across elevations. For example, a mid-elevation peak in flock species richness, size and Shannon's diversity was observed in the Bolivian Andes, as the result of a combination of behavioural and environmental factors [31]. Moreover, MSFs built more connected and cohesive networks in high-elevation sites compared with low-elevation sites [21,23]. However, in this study we found that strong network associations formed at higher elevations only during the breeding season. In the non-breeding season, lower elevations had stronger networks (figure 5). Our contrasting results are consistent with the idea that species tend to forage alone or in conspecific groups if resources are abundant (e.g. wet season in the lower elevations) [35,67] but enhance interspecific associations in stressful environments [21,23]. During the non-breeding (dry) season, the environment may be harsh even at low elevations. For instance, temperature and precipitation are lower during the non-breeding dry season [48], with a concomitant low arthropod biomass and abundance [35,64]. The more connected and cohesive MSFs during the non-breeding (dry) season might arise as a strategy to cope with such environmental conditions [4]. While at higher elevation during the dry season, an even harsher abiotic environment might directly restrict bird distributions and abundances through physiological constraints [7], resulting in less connected and cohesive networks.

Finally, the SEM total effect analysis shows that environmental factors influence flock network structure via both direct and indirect pathways, with season-specific patterns. In the breeding season, effects were concentrated on association strength and connectivity, whereas in the non-breeding season, effects were distributed across multiple network metrics (supplementary material, figure S5). These seasonal differences align with previous studies demonstrating that environmental context and habitat structure can differentially shape interspecific associations and social network patterns [4,21,23].

## 5. Conclusions and conservation implications

Our study found that there were larger and more complex MSF networks in the non-breeding season than in the breeding season. We argue that network structure was primarily affected by reproductive behaviours in the breeding season, but by morphology in the non-breeding season. Specifically, networks dominated by species with larger clutch sizes showed stronger average associations but lower connectivity and cohesion during the breeding season, whereas networks dominated by smaller-bodied species were more cohesive during the non-breeding season. As far as we are aware, ours is the first study revealing how different traits regulate participation in MSFs as seasonality changes. Additionally, our study showed which and how much environmental variables influenced network structure across seasons. Network connectivity was enhanced at higher elevations in the breeding season, but MSFs formed more connected and cohesive networks at low elevations in the non-breeding season. Environmental factors shape networks seasonally, focusing on association strength and connectivity in the breeding season and spanning multiple metrics in the non-breeding season. These findings demonstrate that the interactions between seasonal environmental changes and reproductive behaviours simultaneously determine the interspecific associations of MSFs.

However, we need to acknowledge several potential limitations of this study. For example, we used multiple environmental factors to indirectly represent bird food resources and habitat quality but did not measure them directly. Also, we proposed some potential mechanisms based on our findings, such as reproductive behaviours during the breeding season possibly being the primary factor driving differences in interspecific associations within MSFs, but more field evidence is needed to support this inference, especially focusing on MSF home ranges and movement studies in different seasons.

Our findings have several important conservation implications: (i) conserving the integrity of mountain ecosystems along the entire elevational gradient is vital to preserve habitat and avian biodiversity, particularly in providing seasonally suitable habitats for bird communities, (ii) management strategies should consider the seasonal shifts in network structure, ensuring that both breeding and non-breeding habitat requirements are met, and (iii) protecting key structural habitat features that influence interspecific associations, such as canopy cover and vegetation complexity, can help maintain the connectivity and cohesion of MSFs, supporting stable and resilient bird communities. Together, these insights can guide more effective, seasonally informed conservation planning for mountain bird assemblages.

## Supplementary Material



## Data Availability

Data and R scripts are available from figshare [69]. Supplementary material is available online [70].
