# Supplementary material for: Season-dependent trait–environment filtering influences interaction networks in mixed-species bird flocks
Source: Proc Biol Sci. 2026 Oct 28;293(2081):20261365. doi: 10.1098/rspb.2026.1365 (PMC13725534; doi:10.1098/rspb.2026.1365)
Supplement: Supplementary file 1 [file rspb.2026.1365.zip › rspb.2026.1365/rspb20261365_si_001.pdf]

Electronic Supplementary Material

Season-dependent trait-environment filtering influences interaction networks in mixed-species bird flocks

Yong Shen[1], Zufei Shu[2], Yingming Zhang[2], Sergio Estrada-Villegas[3,4], Eben Goodale[5], Frederick H. Sheldon[6], Qing Quan[1], Fasheng Zou[1], Fuchen Luan[2], Hairong Tan[2], Qiang Zhang[1]*

[1]Guangdong Key Laboratory of Animal Conservation and Resource Utilization, Institute of Zoology, Guangdong Academy of Sciences, Guangzhou 510260, China

[2]Guangdong Chebaling National Nature Reserve, Shaoguan 512526, China

[3]Department of Biology, Faculty of Natural Sciences, Universidad del Rosario, Bogotá 111711, Colombia

[4]Smithsonian Tropical Research Institute, Ancón, Panama

[5]Department of Health and Environmental Science, Xi'an Jiaotong-Liverpool University, Suzhou 215123, China

[6]Museum of Natural Science and Department of Biological Sciences, Louisiana State University, Baton Rouge, LA 70803, USA

Authors for correspondence: Prof. Qiang Zhang; E-mail: zhangqiang06@giz.gd.cn

Journal: *Proceedings of the Royal Society B: Biological Sciences*

DOI: https://doi.org/10.6084/m9.figshare.c.8631041

Supplementary methods

Species-level metrics

For species-level metrics, we calculated closeness centrality, which scores each species based on its proximity to all other species within the network [1]. Interactions between species were also determined relative to the network modules through two metrics: the z-score quantifies how well-connected a species is to other species within its own module; higher values indicate within-module hubs. The participation coefficient ranges from 0 to 1 and describes how evenly a species' links are distributed among modules, with values near 0 indicating links concentrated within its own module and values near 1 indicating links distributed across modules [2].

Supplementary figures and tables

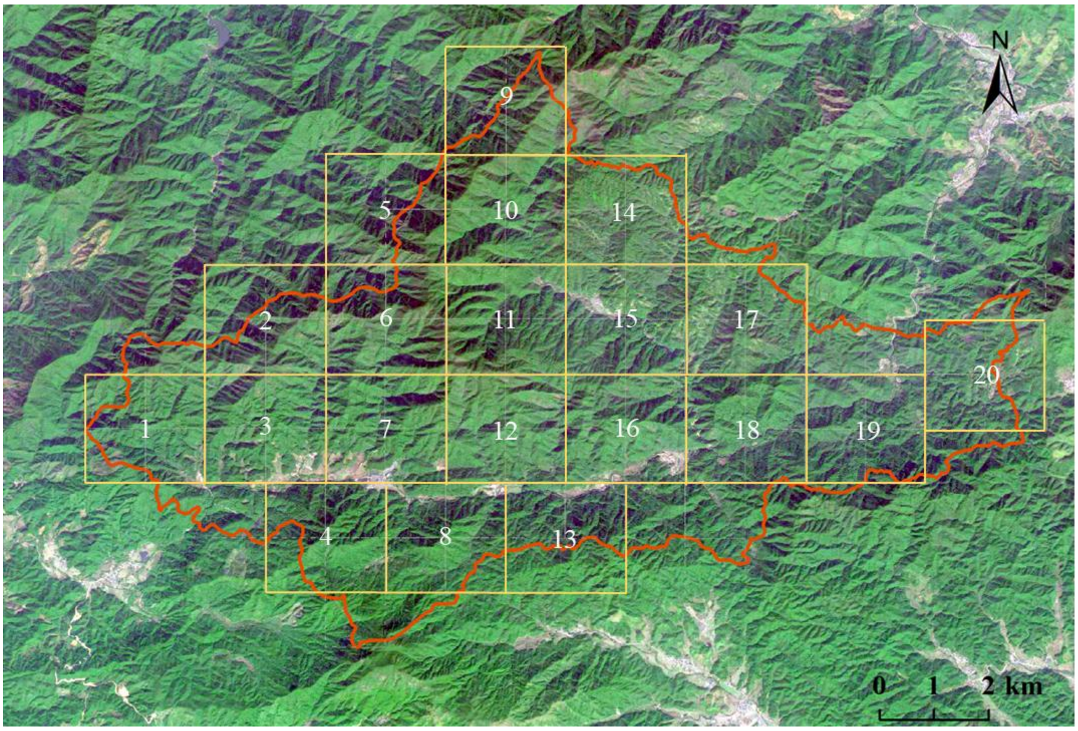

FIGURE S1. The 20 grid cells (each 2 km × 2 km) of bird survey at Chebaling National Nature Reserve. Red and yellow lines indicate boundary of nature reserve and grid cells, respectively.

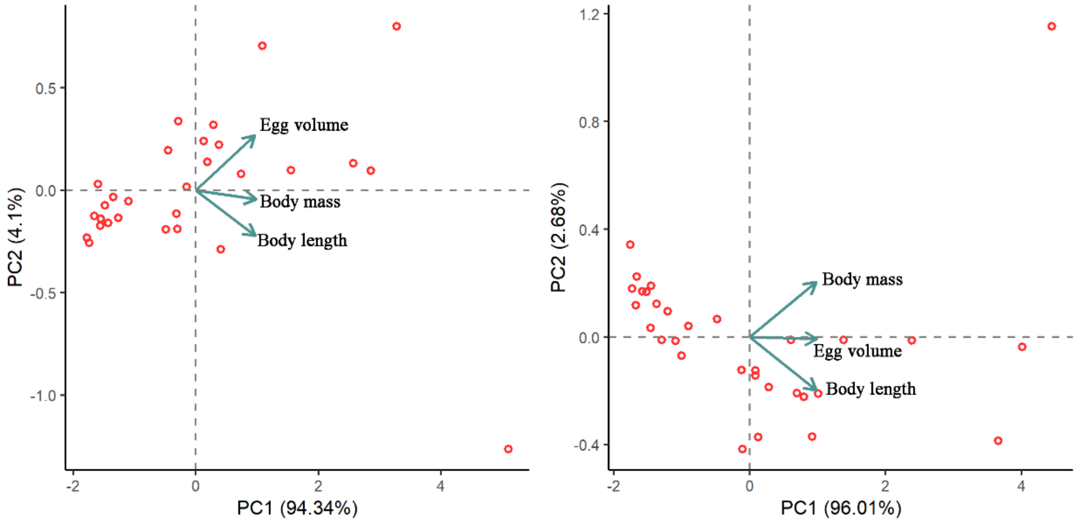

FIGURE S2. Trait loadings biplot of PCA on body length, body mass and egg volume in the breeding (a) and non-breeding season (b).

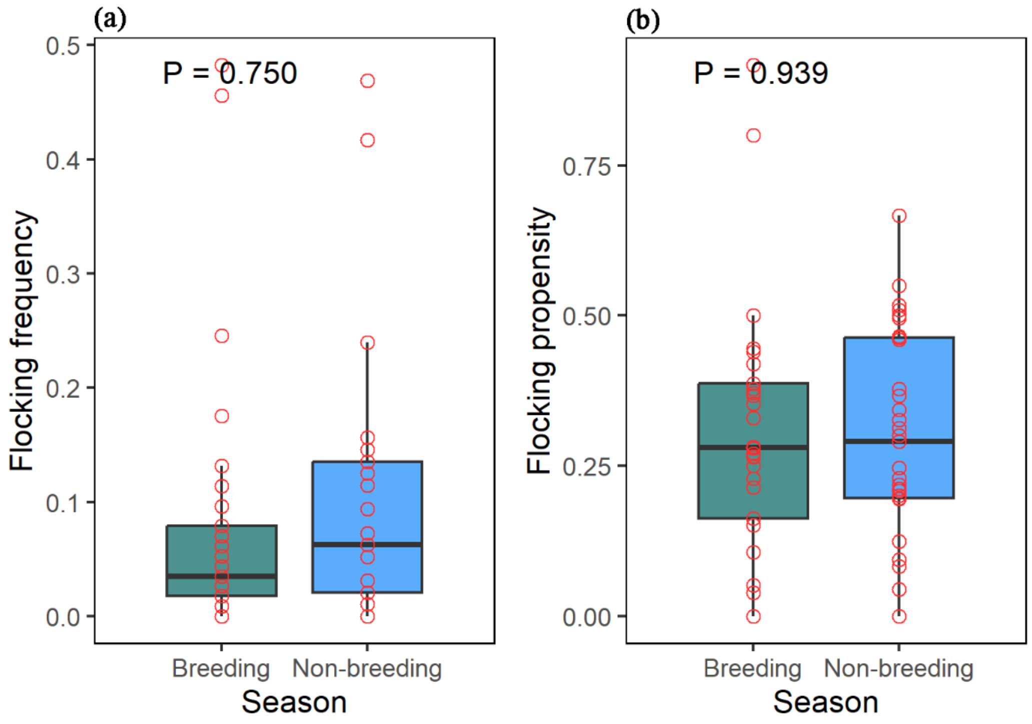

FIGURE S3. Flocking frequency (a) and propensity (b) of species in breeding and non-breeding seasons. Red circles are observational species' flocking frequencies and propensities. P values were obtained from linear mixed-effects ANOVA, with season as the independent variable and species as random effect.

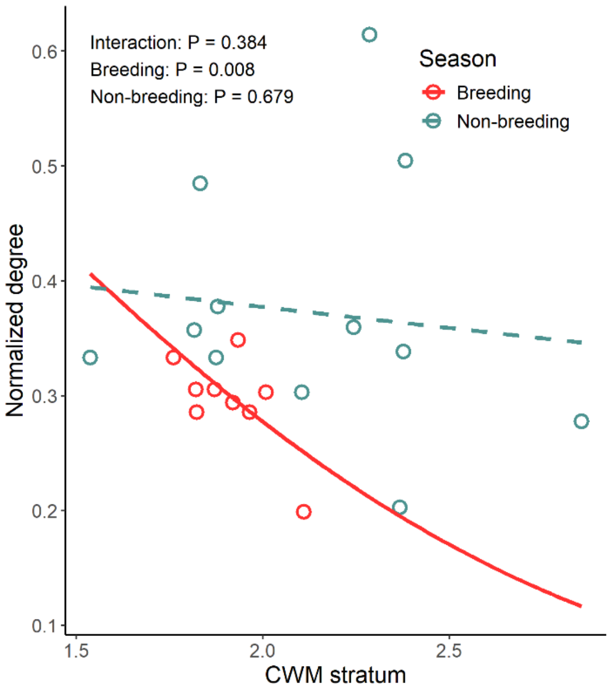

FIGURE S4. Relationships between community-weighted mean (CWM) values of stratum and network-level metrics of mixed-species bird flocks (MSFs) across breeding and non-breeding seasons. Lines and P values are derived from partial regression models that include interaction terms between season and the predictor variable. Solid lines indicate significant relationships ($P < 0.05$), and dashed lines indicate non-significant relationships. Red lines and points represent the breeding season, while green lines and points represent the non-breeding season. Trait values were standardized.

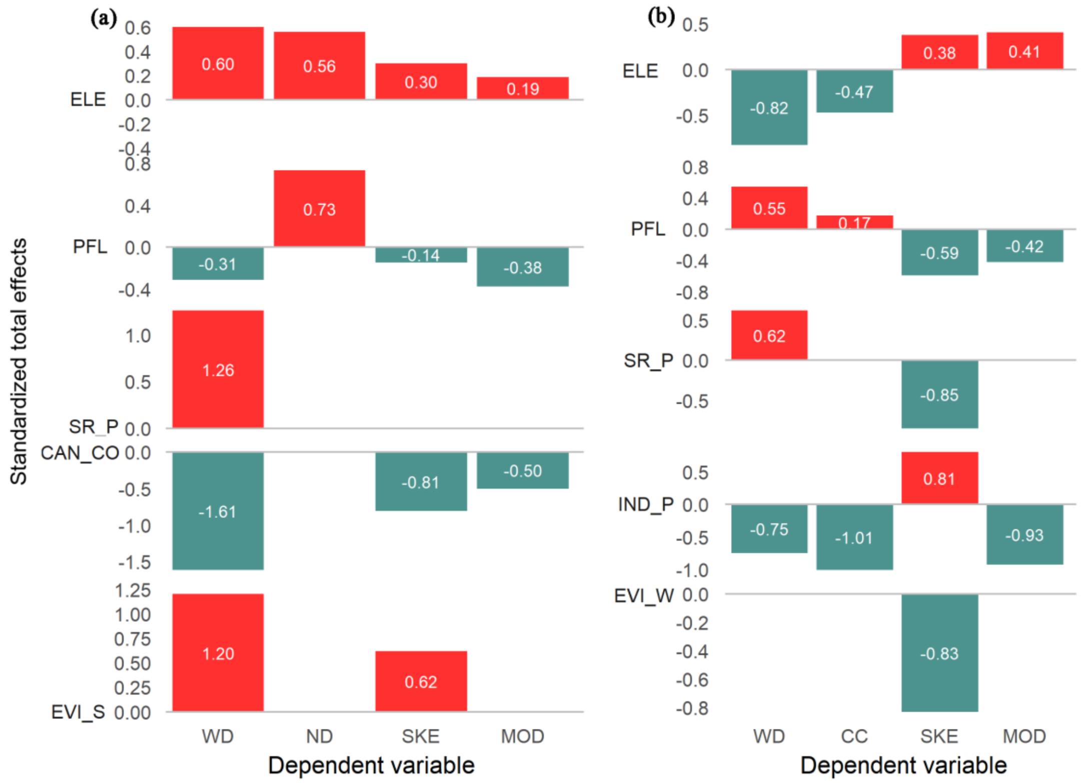

FIGURE S5. Standardized total effects of predictors on network-level metrics of mixed-species bird flocks (MSFs) in the breeding (a) and non-breeding seasons (b). Bars indicate total effects (direct + indirect) of each predictor on each dependent variable. Red bars represent positive effects, and green bars represent negative effects. Only non-zero effects are shown. ELE, elevation; PFL, proportion of forested land; SR_P, species richness of trees; IND_P, number of individual trees; CAN_CO, canopy cover; EVI_S / EVI_W, enhanced vegetation index in summer / winter; WD, weighted degree; ND, normalized degree; CC, clustering coefficient; SKE, skewness of normalized degree; MOD, modularity.

TABLE S1. Summary of network metrics and their interpretation.

Network-level metric	Abbreviation	Definition	Biological interpretation	Level of metric	Reference
Weighted degree	WD	The sum of the simple ratio indexes (SRI) for each node.	Association strength (a higher value indicates stronger association strength). A weighted network is a network in which the ties among nodes have weights assigned to them, which can cooperate in strengthening associations (i.e., edge weights) between the connected species.	Species level, averaged to network level.	[3-5]
Normalized degree	ND	Degree is the number of co-occurrences between each species. The degree is normalized for each species by dividing it by the total number of possible connections.	Connectivity (a high value indicates greater connectivity). Normalized values of average degree can be compared between networks of different sizes (i.e., with different numbers of participating species).	Species level, averaged to network level.	[3, 5, 6]
Density	DEN	The realized proportion of all possible associations. DEN = ND, when using simple ratio indexes to construct networks.	Connectivity (a high value indicates greater connectivity). It describes the overall number of connections contained within a network of flocking species and quantifies them as the proportion of all possible species associations within flocks that are realized.	Network level	[4, 7]
Skewness of ND	SKE	Skewness of the distribution of normalized degrees.	Connectivity (highly negatively skewed distributions reflect networks in which species have larger normalized degree values, i.e., more connections).	Network level.	[5, 7]
Clustering coefficient	CC	The global weighted clustering coefficient represents the extent to which two species, if both connected to a third species, are connected to each other.	Cohesion (a higher value indicates higher cohesion). It describes the extent to which species freely associate within flocks or are excluded due to competition or incompatible foraging behaviours.	Network level.	[5, 7]
Modularity	MOD	Measures how divided the network is into particular subnetworks.	Cohesion (a lower value indicates higher cohesion). It describes how much flocks are partitioned into subgroups within the flocking system.	Network level.	[4, 6-8]

TABLE S2. Correlation matrix among environmental factors for the breeding season using Spearman method. Correlation coefficients and P-values (in parentheses) were shown. Significant relationships were highlighted in italics. See abbreviations in the main text.

Variable	SR_P	IND_P	ELE	EVI_S	PFL
IND_P	-0.226 (0.559)				
ELE	-0.008 (0.983)	0.483 (0.187)			
EVI_S	-0.494 (0.177)	0.033 (0.932)	-0.317 (0.406)		
PFL	-0.084 (0.831)	-0.117 (0.765)	-0.133 (0.732)	*0.750 (0.020)*	
CAN_CO	0.310 (0.417)	-0.250 (0.516)	-0.217 (0.576)	0.567 (0.112)	*0.850 (0.004)*

TABLE S3. Correlation matrix among environmental factors for the non-breeding season using Spearman method. Correlation coefficients and P-values (in parentheses) were shown. Significant relationships were highlighted in italics. See abbreviations in the main text.

Variable	SR_P	IND_P	ELE	EVI_W	PFL
IND_P	0.378 (0.226)				
ELE	0.224 (0.484)	0.399 (0.199)			
EVI_W	-0.510 (0.090)	0.308 (0.331)	-0.007 (0.983)		
PFL	-0.042 (0.897)	-0.497 (0.101)	0.210 (0.513)	-0.161 (0.618)	
CAN_CO	-0.140 (0.665)	*-0.804 (0.002)*	-0.427 (0.167)	-0.168 (0.602)	*0.699 (0.011)*

TABLE S4. The summary of 33 flocking species traits related to body size, diet, foraging stratum and breeding characteristics in this study.

Trait	Unit	Description
Body length	mm	The length from bill to tail
Body mass	g	The body weight of a bird
Egg volume	mm^3	$0.51 \times$ egg length $\times$ egg width^2
Dietary guild	/	Insectivore (1) or omnivore (2)
Vertical stratum	/	Canopy, midstorey or understorey
Clutch size	/	Number of eggs per clutch
Nest type	/	Cavity nest (1) or open nest (2)
Nest location	/	Ground (1), shrub (2) or canopy (3)

TABLE S5. Correlation matrix among continuous traits for breeding season using Spearman method. Correlation coefficients and P-values (in parentheses) were shown. Significant relationships were highlighted in italics.

Variable	Body mass	Body length	Clutch size
Body length	*0.974 (<0.001)*		
Clutch size	-0.081 (0.676)	-0.096 (0.621)	
Egg volume	*0.945 (<0.001)*	*0.938 (<0.001)*	-0.001 (0.995)

TABLE S6. Correlation matrix among continuous traits for non-breeding season using Spearman method. Correlation coefficients and P-values (in parentheses) were shown. Significant relationships were highlighted in italics.

Variable	Body mass	Body length	Clutch size
Body length	*0.966 (<0.001)*		
Clutch size	-0.206 (0.267)	-0.154 (0.408)	
Egg volume	*0.942 (<0.001)*	*0.947 (<0.001)*	-0.088 (0.639)

Table S7. Loadings of bird traits on the first two principal components (PC1 and PC2) for the breeding and non-breeding seasons; higher absolute values indicate stronger contribution of the trait to the respective axis.

	Breeding season		Non-breeding season	
Trait	PC1	PC2	PC1	PC2
Body mass	0.98	-0.04	0.98	0.20
Body length	0.99	-0.22	0.98	-0.20
Egg volume	0.96	0.27	0.99	-0.01

TABLE S8. Summary of mixed-species bird flocks (MSFs) used in this study.

Item	Season	Value
Number of flocks	Breeding	114
	Non-breeding	96
Number of flocking species	Breeding	29
	Non-breeding	31
Total number of individuals in MSFs	Breeding	3121
	Non-breeding	4286
Mean number of species in flocks (Range; SD)	Breeding	2.58 (2-5; 0.80)
	Non-breeding	3.18 (2-13; 1.79)
Mean number of individuals in flocks (Range; SD)	Breeding	27.38 (4-90; 19.72)
	Non-breeding	44.65 (6-277; 45.81)
Mean number of flocks per grid cell (Range; SD)	Breeding	12.67 (7-16; 3.0)
	Non-breeding	8.0 (4-13; 3.64)

98 **TABLE S9.** Flocking frequency and propensity of each species in the breeding and non-breeding seasons.

Species	Frequency_breeding	Frequency_non-breeding	Propensity_breeding	Propensity_non-breeding
Aegithalos concinnus	0.05	0.07	0.28	0.50
Aethopyga christinae	0.03	0.13	0.04	0.20
Alcippe hueti	0.48	0.42	0.28	0.38
Chloropsis hardwickii	0.03	0.03	0.27	0.20
Cyanoderma ruficeps	0.13	0.16	0.39	0.46
Dendrocitta formosae	0.25	0.24	0.28	0.12
Dicaeum ignipectus	0.00	0.02	0.00	0.21
Erpornis zantholeuca	0.08	0.15	0.45	0.51
Erythrogenys gravivox	0.04	0.01	0.21	0.04
Garrulax canorus	0.02	0.00	0.37	0.00
Garrulax monileger	0.10	0.13	0.23	0.34
Garrulax pectoralis	0.18	0.15	0.44	0.29
Harpactes erythrocephalus	0.03	0.01	0.11	0.09
Hemixos castanonotus	0.46	0.47	0.26	0.25
Hypsipetes leucocephalus	0.02	0.24	0.05	0.25
Ixos mcclellandii	0.13	0.07	0.27	0.23
Leiothrix lutea	0.00	0.02	0.00	0.55
Pericrocotus flammeus	0.08	0.06	0.35	0.30
Pericrocotus solaris	0.11	0.14	0.42	0.46
Phyllergates cucullatus	0.01	0.01	0.50	0.50
Phylloscopus castaniceps	0.00	0.02	0.00	0.67
Phylloscopus fuscatus	0.01	0.02	0.50	0.21
Phylloscopus inornatus	0.04	0.03	0.92	0.33

Species	Frequency_breeding	Frequency_non-breeding	Propensity_breeding	Propensity_non-breeding
Phylloscopus proregulus	0.04	0.15	0.50	0.20
Pomatorhinus ruficollis	0.02	0.05	0.15	0.13
Pycnonotus jocosus	0.03	0.05	0.37	0.31
Pycnonotus sinensis	0.02	0.06	0.37	0.52
Spizixos semitorques	0.04	0.03	0.38	0.22
Tarsiger cyanurus	0.00	0.03	0.00	0.08
Tephrodornis virgatus	0.03	0.01	0.25	0.67
Urocissa erythroryncha	0.02	0.00	0.16	0.00
Yuhina castaniceps	0.07	0.09	0.33	0.47
Zosterops japonicus	0.06	0.11	0.80	0.37

Table S10. Seasonal effects on flocking frequency, propensity, MSF sizes and network-level metrics.

Response	Contrast	Estimate	SE	n	P	Marginal R^2	95% CI
Flocking frequency	Breeding vs. Non-breeding	-0.333	1.040	33	0.750	<0.001	-2.449, 1.782
Flocking propensity	Breeding vs. Non-breeding	-0.003	0.045	33	0.939	<0.001	-0.095, 0.088
Number of species in MSFs	Breeding vs. Non-breeding	-0.481	0.236	210	0.044	0.028	-0.949, -0.014
Number of individuals in MSFs	Breeding vs. Non-breeding	-23.200	5.950	210	<0.001	0.096	-34.974, -11.433
Weighted degree	Breeding vs. Non-breeding	-1.250	0.493	21	0.024	0.271	-2.315, -0.193
Normalized degree	Breeding vs. Non-breeding	-0.078	0.042	21	0.048	0.166	-0.169, -0.012
Clustering coefficient	Breeding vs. Non-breeding	-0.247	0.060	21	0.001	0.492	-0.377, -0.118
Skewness	Breeding vs. Non-breeding	0.902	0.215	21	0.007	0.328	0.364, 1.439
Modularity	Breeding vs. Non-breeding	0.027	0.060	21	0.660	0.012	-0.102, 0.157

For flocking frequency and propensity, n indicates the number of species. For MSF-size comparisons, n indicates the number of MSFs. For network-level metrics, n indicates the number of networks. Estimates represent breeding season minus non-breeding season. Negative estimates indicate higher values in the non-breeding season.

TABLE S11. Mean closeness centrality, participation coefficient and z-score for each vertex (species) in the breeding season. High closeness centralities indicate species are closer to all other nodes. Centrality equal to 1 occurred in the subgroups with only two species. Participation coefficient of a species varies between 0 (all interactions are within the module) and 1 (all interactions are uniformly distributed among all modules). High z-score indicates a species is well-connected within its module. Calculation of z-score is not available (NA) when all species connected with each other within its module.

Species	Grid number	Closeness centrality	Participant coefficient	Z-scores
Hemixos castanonotus	4	4.08	0.48	1.50
Dendrocitta formosae	4	3.86	0.32	1.40
Alcippe hueti	4	3.18	0.38	0.53
Spizixos semitorques	4	2.87	0.00	-0.35
Harpactes erythrocephalus	4	2.60	0.00	-1.23
Ixos mcclellandii	4	2.56	0.00	-0.50
Pericrocotus flammeus	4	2.56	0.00	-0.50
Zosterops japonicus	4	2.56	0.00	-0.50
Garrulax pectoralis	4	2.25	0.00	-0.35
Hemixos castanonotus	8	3.94	0.50	1.63
Pericrocotus flammeus	8	3.72	0.56	0.18
Pericrocotus solaris	8	3.36	0.48	0.18
Alcippe hueti	8	3.04	0.44	1.10
Chloropsis hardwickii	8	2.82	0.38	0.18
Aegithalos concinnus	8	2.73	0.00	0.00
Erpornis zantholeuca	8	2.72	0.00	-0.82
Spizixos semitorques	8	2.72	0.00	-0.82

Species	Grid number	Closeness centrality	Participant coefficient	Z-scores
Dendrocitta formosae	8	2.67	0.50	0.00
Ixos mcclellandii	8	2.22	0.50	NA
Cyanoderma ruficeps	8	1.96	0.00	-1.64
Zosterops japonicus	8	1.10	0.00	NA
Alcippe hueti	9	2.50	0.61	0.87
Yuhina castaniceps	9	1.90	0.50	-0.58
Hemixos castanonotus	9	1.78	0.00	-0.87
Phylloscopus proregulus	9	1.78	0.00	-0.87
Cyanoderma ruficeps	9	1.74	0.50	NA
Pomatorhinus ruficollis	9	1.74	0.50	NA
Garrulax monileger	9	1.47	0.00	1.15
Zosterops japonicus	9	1.38	0.00	0.87
Garrulax pectoralis	9	0.89	0.00	-0.58
Alcippe hueti	10	3.17	0.57	0.96
Dendrocitta formosae	10	3.08	0.56	NA
Hemixos castanonotus	10	2.71	0.64	NA
Yuhina castaniceps	10	2.56	0.48	-0.24
Ixos mcclellandii	10	2.54	0.50	NA
Zosterops japonicus	10	2.08	0.00	NA
Garrulax monileger	10	2.05	0.00	NA
Phylloscopus inornatus	10	1.92	0.00	-0.24
Erpornis zantholeuca	10	1.90	0.00	-1.43
Pomatorhinus ruficollis	10	1.77	0.00	NA
Cyanoderma ruficeps	10	1.68	0.00	0.96
Garrulax pectoralis	10	1.55	0.00	NA

Species	Grid number	Closeness centrality	Participant coefficient	Z-scores
Alcippe hueti	11	4.63	0.68	1.55
Hemixos castanonotus	11	4.59	0.74	0.96
Aegithalos concinnus	11	4.48	0.66	0.22
Cyanoderma ruficeps	11	4.05	0.64	1.15
Hypsipetes leucocephalus	11	3.78	0.63	-1.43
Dendrocitta formosae	11	3.74	0.57	0.96
Garrulax pectoralis	11	3.51	0.61	NA
Ixos mcclellandii	11	3.47	0.00	-0.24
Erythrogenys gravivox	11	3.28	0.38	-0.24
Pericrocotus solaris	11	3.26	0.67	-0.58
Aethopyga christinae	11	3.20	0.38	0.22
Garrulax monileger	11	3.11	0.44	NA
Tephrodornis virgatus	11	2.99	0.44	NA
Harpactes erythrocephalus	11	2.86	0.00	-1.11
Zosterops japonicus	11	2.86	0.00	0.22
Erpornis zantholeuca	11	2.07	0.00	-1.11
Pycnonotus jocosus	11	1.88	0.50	-0.58
Hemixos castanonotus	13	6.01	0.41	1.48
Erpornis zantholeuca	13	5.15	0.00	-0.30
Dendrocitta formosae	13	4.71	0.48	0.30
Pericrocotus flammeus	13	4.36	0.44	NA
Pericrocotus solaris	13	4.36	0.44	NA
Alcippe hueti	13	4.31	0.00	1.48
Garrulax pectoralis	13	3.80	0.00	-0.30
Harpactes erythrocephalus	13	3.77	0.00	-0.89

Species	Grid number	Closeness centrality	Participant coefficient	Z-scores
Ixos mcclellandii	13	3.77	0.00	-0.89
Garrulax monileger	13	2.91	0.00	-0.89
Alcippe hueti	14	4.40	0.53	1.34
Zosterops japonicus	14	3.70	0.00	0.00
Yuhina castaniceps	14	3.69	0.00	-0.45
Erythrogenys gravivox	14	3.56	0.48	1.22
Garrulax monileger	14	3.35	0.00	-0.45
Cyanoderma ruficeps	14	3.19	0.47	0.45
Garrulax pectoralis	14	3.03	0.00	0.45
Phyllergates cucullatus	14	2.91	0.50	NA
Phylloscopus fuscatus	14	2.91	0.50	NA
Phylloscopus proregulus	14	2.91	0.00	-1.34
Spizixos semitorques	14	2.91	0.50	-1.22
Hemixos castanonotus	14	2.81	0.00	1.34
Chloropsis hardwickii	14	2.00	0.44	0.00
Phylloscopus inornatus	14	2.00	0.44	0.00
Ixos mcclellandii	14	1.85	0.00	-1.34
Alcippe hueti	15	5.80	0.75	0.24
Hemixos castanonotus	15	4.51	0.57	1.43
Pericrocotus flammeus	15	3.74	0.48	NA
Garrulax pectoralis	15	3.60	0.48	1.22
Ixos mcclellandii	15	3.47	0.00	-0.96
Aegithalos concinnus	15	3.28	0.44	NA
Aethopyga christinae	15	3.28	0.44	0.00
Erythrogenys gravivox	15	3.28	0.44	0.00

Species	Grid number	Closeness centrality	Participant coefficient	Z-scores
Garrulax canorus	15	3.28	0.44	NA
Phylloscopus inornatus	15	3.28	0.44	NA
Spizixos semitorques	15	3.28	0.44	NA
Cyanoderma ruficeps	15	2.89	0.38	0.24
Erpornis zantholeuca	15	2.56	0.00	-0.96
Garrulax monileger	15	2.45	0.50	-1.22
Phylloscopus proregulus	15	2.28	0.44	NA
Hemixos castanonotus	16	2.86	0.72	0.00
Pericrocotus solaris	16	2.77	0.45	1.48
Alcippe hueti	16	2.52	0.50	0.89
Tephrodornis virgatus	16	2.37	0.56	0.18
Dendrocitta formosae	16	2.27	0.44	0.89
Garrulax pectoralis	16	2.22	0.67	NA
Pycnonotus sinensis	16	2.15	0.50	0.18
Phylloscopus proregulus	16	2.14	0.00	0.30
Erpornis zantholeuca	16	2.08	0.56	1.22
Pericrocotus flammeus	16	1.93	0.00	-0.89
Yuhina castaniceps	16	1.93	0.00	-0.89
Urocissa erythroryncha	16	1.86	0.50	-0.89
Phylloscopus inornatus	16	1.71	0.44	0.00
Pycnonotus jocosus	16	1.52	0.00	1.10
Aegithalos concinnus	16	1.48	0.50	-1.22
Ixos mcclellandii	16	1.48	0.00	-0.89
Garrulax canorus	16	1.31	0.00	NA
Hypsipetes leucocephalus	16	1.20	0.00	0.18

Species	Grid number	Closeness centrality	Participant coefficient	*Z*-scores
Spizixos semitorques	16	0.88	0.00	-1.64

TABLE S12. Mean closeness centrality, participation coefficient and z-score for each vertex (species) in the non-breeding season. High closeness centralities indicate species are closer to all other nodes. Centrality equal to 1 occurred in the subgroups with only two species. Participation coefficient of a species varies between 0 (all interactions are within the module) and 1 (all interactions are uniformly distributed among all modules). High z-score indicates a species is well-connected within its module. Calculation of z-score is not available (NA) when all species connected with each other within its module.

Species	Grid number	Closeness centrality	Participant coefficient	Z-scores
Alcippe hueti	1	3.38	0.24	1.34
Hemixos castanonotus	1	3.23	0.49	0.17
Dendrocitta formosae	1	2.83	0.00	0.17
Zosterops japonicus	1	2.80	0.00	0.75
Garrulax pectoralis	1	2.50	0.50	NA
Hypsipetes leucocephalus	1	2.36	0.50	-0.58
Phylloscopus proregulus	1	2.29	0.00	-1.01
Cyanoderma ruficeps	1	2.25	0.00	0.17
Pomatorhinus ruficollis	1	2.24	0.00	-1.59
Pericrocotus flammeus	1	2.22	0.50	-0.58
Pericrocotus solaris	1	1.84	0.44	1.15
Garrulax monileger	1	1.17	0.00	NA
Dendrocitta formosae	2	2.64	0.56	NA
Alcippe hueti	2	2.15	0.38	1.22
Garrulax pectoralis	2	1.92	0.50	NA
Hemixos castanonotus	2	1.72	0.44	0.00
Zosterops japonicus	2	1.68	0.44	0.00

Species	Grid number	Closeness centrality	Participant coefficient	Z-scores
Hypsipetes leucocephalus	2	1.51	0.00	NA
Cyanoderma ruficeps	2	1.33	0.00	-1.22
Garrulax monileger	2	1.05	0.00	NA
Hemixos castanonotus	3	2.00	0.00	1.22
Garrulax monileger	3	1.20	0.00	-1.22
Pycnonotus sinensis	3	1.20	0.00	0.00
Spizixos semitorques	3	1.20	0.00	0.00
Alcippe hueti	3	1.00	0.00	NA
Erpornis zantholeuca	3	1.00	0.00	NA
Pycnonotus sinensis	7	2.00	0.00	0.96
Zosterops japonicus	7	2.00	0.00	0.96
Aethopyga christinae	7	1.71	0.00	-0.24
Dicaeum ignipectus	7	1.71	0.00	-0.24
Pycnonotus jocosus	7	1.50	0.00	-1.43
Dendrocitta formosae	7	1.00	0.00	NA
Garrulax monileger	7	1.00	0.00	NA
Garrulax pectoralis	7	1.00	0.00	NA
Hemixos castanonotus	7	1.00	0.00	NA
Alcippe hueti	10	4.26	0.61	NA
Dendrocitta formosae	10	3.36	0.56	0.18
Hemixos castanonotus	10	3.35	0.56	0.18
Hypsipetes leucocephalus	10	3.30	0.63	NA
Garrulax monileger	10	2.61	0.50	NA
Garrulax pectoralis	10	2.61	0.50	NA
Erpornis zantholeuca	10	2.57	0.50	-1.64

Species	Grid number	Closeness centrality	Participant coefficient	Z-scores
Aegithalos concinnus	10	2.45	0.00	1.10
Phylloscopus proregulus	10	2.05	0.00	0.18
Leiothrix lutea	10	1.91	0.00	NA
Hemixos castanonotus	11	2.33	0.57	0.73
Aethopyga christinae	11	2.09	0.44	NA
Alcippe hueti	11	2.05	0.50	NA
Dendrocitta formosae	11	1.91	0.63	NA
Pycnonotus jocosus	11	1.71	0.00	0.73
Pycnonotus sinensis	11	1.71	0.00	0.73
Hypsipetes leucocephalus	11	1.61	0.38	-1.10
Ixos mcclellandii	11	1.52	0.00	-1.10
Cyanoderma ruficeps	11	1.12	0.00	NA
Erpornis zantholeuca	11	1.12	0.00	NA
Zosterops japonicus	11	1.12	0.00	NA
Pericrocotus flammeus	11	1.02	0.00	NA
Yuhina castaniceps	11	1.02	0.00	NA
Garrulax monileger	11	1.00	0.00	NA
Garrulax pectoralis	11	1.00	0.00	NA
Pericrocotus solaris	11	1.00	0.00	NA
Phyllergates cucullatus	11	1.00	0.00	NA
Phylloscopus proregulus	11	1.00	0.00	NA
Aegithalos concinnus	13	2.25	0.00	NA
Alcippe hueti	13	2.25	0.00	NA
Cyanoderma ruficeps	13	2.25	0.00	NA
Pericrocotus solaris	13	2.25	0.00	NA

Species	Grid number	Closeness centrality	Participant coefficient	Z-scores
Erpornis zantholeuca	13	1.00	0.00	NA
Garrulax monileger	13	1.00	0.00	NA
Garrulax pectoralis	13	1.00	0.00	NA
Ixos mcclellandii	13	1.00	0.00	NA
Phylloscopus inornatus	13	1.00	0.00	NA
Phylloscopus proregulus	13	1.00	0.00	NA
Pomatorhinus ruficollis	13	1.00	0.00	NA
Tarsiger cyanurus	13	1.00	0.00	NA
Hemixos castanonotus	15	4.18	0.52	1.36
Dendrocitta formosae	15	3.05	0.50	-0.58
Alcippe hueti	15	3.01	0.47	0.94
Pericrocotus flammeus	15	3.00	0.00	-0.34
Phylloscopus castaniceps	15	3.00	0.00	-0.34
Erythrogenys gravivox	15	2.97	0.32	-0.77
Hypsipetes leucocephalus	15	2.93	0.00	-0.34
Aethopyga christinae	15	2.91	0.44	NA
Phylloscopus proregulus	15	2.91	0.44	NA
Yuhina castaniceps	15	2.91	0.44	NA
Aegithalos concinnus	15	2.91	0.44	NA
Pycnonotus jocosus	15	2.68	0.00	-2.04
Erpornis zantholeuca	15	2.38	0.00	0.51
Pericrocotus solaris	15	2.34	0.20	0.94
Cyanoderma ruficeps	15	2.31	0.49	NA
Ixos mcclellandii	15	2.26	0.24	0.09
Spizixos semitorques	15	1.66	0.00	1.15

Species	Grid number	Closeness centrality	Participant coefficient	Z-scores
Pomatorhinus ruficollis	15	0.93	0.00	-0.58
Hemixos castanonotus	17	4.35	0.60	1.15
Aethopyga christinae	17	3.91	0.49	NA
Alcippe hueti	17	3.72	0.50	NA
Erpornis zantholeuca	17	3.53	0.50	NA
Pericrocotus solaris	17	3.30	0.50	-0.86
Hypsipetes leucocephalus	17	3.17	0.58	0.48
Cyanoderma ruficeps	17	3.12	0.44	-0.19
Phylloscopus inornatus	17	3.08	0.00	-1.53
Phylloscopus proregulus	17	3.03	0.48	1.15
Leiothrix lutea	17	2.76	0.50	NA
Zosterops japonicus	17	2.75	0.57	-0.19
Yuhina castaniceps	17	2.68	0.47	NA
Dendrocitta formosae	17	2.31	0.38	NA
Chloropsis hardwickii	17	2.20	0.00	NA
Pomatorhinus ruficollis	17	2.20	0.00	NA
Hypsipetes leucocephalus	18	2.61	0.73	NA
Alcippe hueti	18	2.40	0.73	NA
Aethopyga christinae	18	2.34	0.63	NA
Hemixos castanonotus	18	2.34	0.63	NA
Aegithalos concinnus	18	2.22	0.63	NA
Chloropsis hardwickii	18	2.22	0.63	NA
Pericrocotus solaris	18	2.22	0.63	NA
Phylloscopus castaniceps	18	2.22	0.63	NA
Tephrodornis virgatus	18	2.22	0.63	NA

Species	Grid number	Closeness centrality	Participant coefficient	Z-scores
Dendrocitta formosae	18	2.14	0.69	NA
Pericrocotus flammeus	18	2.14	0.69	NA
Phylloscopus proregulus	18	2.14	0.69	NA
Dicaeum ignipectus	18	2.12	0.50	NA
Zosterops japonicus	18	2.09	0.63	NA
Erpornis zantholeuca	18	1.96	0.28	NA
Yuhina castaniceps	18	1.96	0.28	NA
Garrulax monileger	18	1.00	0.00	NA
Garrulax pectoralis	18	1.00	0.00	NA
Hemixos castanonotus	19	4.04	0.50	1.51
Alcippe hueti	19	3.39	0.41	0.17
Yuhina castaniceps	19	2.91	0.28	0.17
Dendrocitta formosae	19	2.82	0.00	-0.50
Cyanoderma ruficeps	19	2.74	0.00	-1.85
Phylloscopus fuscatus	19	2.67	0.00	-0.50
Aegithalos concinnus	19	2.62	0.28	NA
Chloropsis hardwickii	19	2.62	0.28	NA
Harpactes erythrocephalus	19	2.62	0.28	NA
Pericrocotus solaris	19	2.62	0.28	NA
Phylloscopus inornatus	19	2.62	0.28	NA
Phylloscopus proregulus	19	2.62	0.28	NA
Tarsiger cyanurus	19	2.62	0.00	0.84
Hypsipetes leucocephalus	19	2.57	0.00	0.17
Ixos mcclellandii	19	2.36	0.50	NA
Garrulax pectoralis	19	2.32	0.44	NA

Species	Grid number	Closeness centrality	Participant coefficient	Z-scores
Garrulax monileger	19	1.15	0.00	NA
Dendrocitta formosae	20	2.06	0.48	NA
Hemixos castanonotus	20	1.57	0.32	1.10
Alcippe hueti	20	1.50	0.00	NA
Cyanoderma ruficeps	20	1.50	0.00	NA
Erpornis zantholeuca	20	1.14	0.00	NA
Hypsipetes leucocephalus	20	1.14	0.00	NA
Pericrocotus flammeus	20	1.14	0.38	0.18
Phylloscopus fuscatus	20	1.14	0.38	0.18
Phylloscopus proregulus	20	1.14	0.00	NA
Pomatorhinus ruficollis	20	1.14	0.00	NA
Yuhina castaniceps	20	1.14	0.38	0.18
Aethopyga christinae	20	0.92	0.00	-1.64

Table S13. Regression models for trait and network-level metric relationships.

Season	Network metric	Trait predictor	Model family	Estimate	SE	n networks	P	R^2	95% CI
Breeding	Weighted degree	Clutch size	GLM-Gamma (link = "log")	2.649	0.891	9	0.021	0.501	0.776, 4.661
Non-breeding	Weighted degree	Clutch size	GLM-Gamma (link = "log")	-0.586	1.732	12	0.742	0.015	-3.453, 2.454
Breeding	Skewness	Clutch size	GLM-Gaussian	3.442	1.775	9	0.045	0.257	0.756, 7.640
Non-breeding	Skewness	Clutch size	GLM-Gaussian	1.019	1.937	12	0.610	0.027	-3.297, 5.335
Breeding	Modularity	Clutch size	Beta regression	3.496	1.096	9	0.001	0.540	1.347, 5.644
Non-breeding	Modularity	Clutch size	Beta regression	1.056	1.510	12	0.484	0.044	-1.904, 4.016
Breeding	Weighted degree	CWM PC1	GLM-Gamma (link = "log")	0.042	0.287	9	0.888	0.003	-0.466, 0.601
Non-breeding	Weighted degree	CWM PC1	GLM-Gamma (link = "log")	-0.842	0.365	12	0.044	0.361	-1.488, -0.101
Breeding	Clustering coefficient	CWM PC1	Beta regression	0.227	0.230	9	0.324	0.096	-0.223, 0.677
Non-breeding	Clustering coefficient	CWM PC1	Beta regression	-1.024	0.391	12	0.009	0.344	-1.790, -0.257

R^2 values represent deviance-based pseudo-R^2 for Gamma GLMs, pseudo-R^2 for beta regression models, and ordinary R^2 for Gaussian models.

Table S14. Season × trait interaction models for trait and network-level metric relationships.

Network metric	Trait	Term	Estimate	SE	n networks	P	R^2	95% CI
Weighted degree	Clutch size	Trait	2.649	1.659	21	0.129		-0.739, 6.508
Weighted degree	Clutch size	Season	0.100	0.566	21	0.862	0.485	-1.087, 1.183
Weighted degree	Clutch size	Trait × Season	-3.235	2.180	21	0.156		-7.734, 0.970
Skewness	Clutch size	Trait	3.442	2.081	21	0.116		-0.948, 7.832
Skewness	Clutch size	Season	-1.367	0.710	21	0.071	0.287	-2.866, 0.131
Skewness	Clutch size	Trait × Season	-2.423	2.735	21	0.388		-8.194, 3.348
Modularity	Clutch size	Trait	3.398	1.538	21	0.027		0.384, 6.411
Modularity	Clutch size	Season	-0.682	0.517	21	0.188	0.229	-1.696, 0.332
Modularity	Clutch size	Trait × Season	-2.320	2.016	21	0.250		-6.271, 1.631
Weighted degree	CWM PC1	Trait	0.042	0.368	21	0.911		-0.603, 0.770
Weighted degree	CWM PC1	Season	0.824	0.226	21	0.002	0.573	0.370, 1.255
Weighted degree	CWM PC1	Trait × Season	-0.884	0.490	21	0.089		-1.81, 0.030
Clustering coefficient	CWM PC1	Trait	0.173	0.145	21	0.235		-0.112, 0.458
Clustering coefficient	CWM PC1	Season	0.474	0.167	21	0.005	0.601	0.147, 0.801
Clustering coefficient	CWM PC1	Trait × Season	-0.543	0.238	21	0.022		-1.008, -0.077

The interaction models used all 21 networks, including 9 breeding-season networks and 12 non-breeding-season networks. R^2 is shown once per model.

Table S15. SEM model fit and final model structure.

Season	Final model structure	AIC	BIC	Fisher's C	df	P
Breeding	WD ~ EVI_S + CAN_CO + SR_P; ND ~ ELE + PFL; SKE ~ EVI_S + CAN_CO; MOD ~ CAN_CO; EVI_S ~ PFL; CAN_CO ~ ELE + PFL	93.625	98.161	47.625	44	0.327
Non-breeding	WD ~ ELE + SR_P + IND_P; CC ~ PFL + IND_P; SKE ~ EVI_W + SR_P + IND_P; MOD ~ ELE + PFL + IND_P; CAN_CO ~ ELE + PFL; IND_P ~ ELE + PFL	110.758	123.850	56.758	48	0.181

ELE, elevation; PFL, proportion of forested land; SR_P, tree species richness; IND_P, number of individual trees; CAN_CO, canopy cover; EVI_S/EVI_W, enhanced vegetation index in summer/winter; WD, weighted degree; ND, normalized degree; CC, clustering coefficient; SKE, skewness of normalized degree; MOD, modularity.

Table S16. SEM direct path coefficients.

Season	Response	Predictor	Estimate	SE	P	R^2 response	95% CI
Breeding	WD	EVI_S	1.204	0.586	0.095	0.58	0.056, 2.352
Breeding	WD	CAN_CO	-1.614	0.622	0.048	0.58	-2.832, -0.396
Breeding	WD	SR_P	1.261	0.600	0.090	0.58	0.084, 2.437
Breeding	ND	ELE	0.562	0.178	0.020	0.81	0.214, 0.910
Breeding	ND	PFL	0.732	0.178	0.006	0.81	0.384, 1.081
Breeding	SKE	EVI_S	0.618	0.302	0.086	0.57	0.027, 1.210
Breeding	SKE	CAN_CO	-0.809	0.302	0.037	0.57	-1.400, -0.218
Breeding	MOD	CAN_CO	-0.501	0.327	0.170	0.25	-1.142, 0.141
Breeding	EVI_S	PFL	0.746	0.252	0.021	0.56	0.253, 1.239
Breeding	CAN_CO	ELE	-0.374	0.213	0.129	0.73	-0.790, 0.043
Breeding	CAN_CO	PFL	0.750	0.213	0.012	0.73	0.333, 1.166
Non-breeding	WD	ELE	-0.474	0.186	0.034	0.76	-0.837, -0.110
Non-breeding	WD	SR_P	0.624	0.203	0.015	0.76	0.227, 1.021
Non-breeding	WD	IND_P	-0.746	0.205	0.007	0.76	-1.148, -0.344
Non-breeding	CC	PFL	-0.567	0.278	0.072	0.60	-1.112, -0.022
Non-breeding	CC	IND_P	-1.010	0.278	0.006	0.60	-1.555, -0.465
Non-breeding	SKE	EVI_W	-0.831	0.329	0.035	0.50	-1.474, -0.187
Non-breeding	SKE	SR_P	-0.848	0.376	0.054	0.50	-1.585, -0.112
Non-breeding	SKE	IND_P	0.808	0.338	0.044	0.50	0.146, 1.470
Non-breeding	MOD	ELE	0.841	0.286	0.019	0.60	0.280, 1.401
Non-breeding	MOD	PFL	-1.098	0.354	0.015	0.60	-1.793, -0.402
Non-breeding	MOD	IND_P	-0.926	0.370	0.037	0.60	-1.652, -0.201

Season	Response	Predictor	Estimate	SE	P	R^2 response	95% CI
Non-breeding	CAN_CO	ELE	-0.445	0.193	0.046	0.68	-0.822, -0.067
Non-breeding	CAN_CO	PFL	0.777	0.193	0.003	0.68	0.399, 1.154
Non-breeding	IND_P	ELE	0.466	0.206	0.049	0.63	0.063, 0.869
Non-breeding	IND_P	PFL	-0.733	0.206	0.006	0.63	-1.136, -0.330

ELE, elevation; PFL, proportion of forested land; SR_P, tree species richness; IND_P, number of individual trees; CAN_CO, canopy cover; EVI_S/EVI_W, enhanced vegetation index in summer/winter; WD, weighted degree; ND, normalized degree; CC, clustering coefficient; SKE, skewness of normalized degree; MOD, modularity. Estimates are standardized coefficients.

REFERENCES

1. Montaño-Centellas FA. 2020 Interaction networks of avian mixed-species flocks along elevation in the tropical Andes. Ecography 43, 930-942. (doi:10.1111/ecog.05135)
2. Delmas E et al. 2019 Analysing ecological networks of species interactions. Biol. Rev. 94, 16-36. (doi:10.1111/brv.12433)
3. Zhou L, Mammides C, Chen Y, Zhou W, Dai W, Braun EL, Kimball RT, Liu Y, Robinson SK & Goodale E. 2022 High association strengths are linked to phenotypic similarity, including plumage color and patterns, of participants in mixed-species bird flocks of southwestern China. Curr. Zool. zoac096. (doi: 10.1093/cz/zoac096)
4. Shen Y, Holyoak M, Goodale E, Mammides C, Zou F, Chen Y, Zhang C, Quan Q & Zhang Q. 2022 Mixed-species bird flocks re-assemble interspecific associations across an elevational gradient. Proc. R. Soc. B 289, 20221840. (doi: 10.1098/rspb.2022.1840)
5. Mokross K, Ryder TB, Côrtes MC, Wolfe JD & Stouffer PC. 2014 Decay of interspecific avian flock networks along a disturbance gradient in Amazonia. Proc. R. Soc. B 281, 20132599. (doi: 10.1098/rspb.2013.2599)
6. Montaño-Centellas FA et al. 2023 Network structure of avian mixed-species flocks decays with elevation and latitude across the Andes. Philos. Trans. R. Soc. B 378, 20220099. (doi: 10.1098/rstb.2022.0099)
7. Montaño-Centellas FA. 2020 Interaction networks of avian mixed-species flocks along elevation in the tropical Andes. Ecography 43, 930-942. (doi: 10.1111/ecog.05135)
8. Clauset A, Newman MEJ & Moore C. 2004 Finding community structure in very large networks. Phys. Rev. E 70, 066111. (doi: 10.1103/PhysRevE.70.066111)
